# Lanthanide DO3A-Complexes Bearing Peptide Substrates: The Effect of Peptidic Side Chains on Metal Coordination and Relaxivity

**DOI:** 10.3390/molecules26082176

**Published:** 2021-04-09

**Authors:** Sophie Laine, Jean-François Morfin, Mathieu Galibert, Vincent Aucagne, Célia S. Bonnet, Éva Tóth

**Affiliations:** Centre de Biophysique Moléculaire, CNRS UPR 4301, Rue Charles Sadron, CEDEX 2, 45071 Orléans, France; laine-sophie@laposte.net (S.L.); jmorfin@cnrs-orleans.fr (J.-F.M.); mathieu.galibert@genepep.com (M.G.); vincent.aucagne@cnrs-orleans.fr (V.A.); celia.bonnet@cnrs.fr (C.S.B.)

**Keywords:** lanthanide, MRI contrast agents, peptide, enzymatic detection

## Abstract

Two DO3A-type ligands conjugated to substrates of urokinase (L3) and caspase-3 (L4) via a propyl-amide linker were synthesized and their lanthanide(III) (Ln^3+^) complexes studied. A model compound without peptide substrate (L2) and an amine derivative ligand mimicking the state after enzymatic cleavage (L1) were also prepared. Proton Nuclear Magnetic Relaxation Dispersion (NMRD) profiles recorded on the gadolinium(III) (Gd^3+^) complexes, complemented with the assessment of hydration numbers via luminescence lifetime measurements on the Eu^3+^ analogues, allowed us to characterize the lanthanide coordination sphere in the chelates. These data suggest that the potential donor groups of the peptide side chains (carboxylate, amine) interfere in metal coordination, leading to non-hydrated LnL3 and LnL4 complexes. Nevertheless, GdL3 and GdL4 retain a relatively high relaxivity due to an important second-sphere contribution generated by the strongly hydrophilic peptide chain. Weak PARACEST effects are detected for the amine-derivative EuL1 and NdL1 chelates. Unfortunately, the GdL3 and GdL4 complexes are not significantly converted by the enzymes. The lack of enzymatic recognition of these complexes can likely be explained by the participation of donor groups from the peptide side chain in metal coordination.

## 1. Introduction

Activity-based diagnostics refers to in vitro detection or in vivo imaging of disease-specific catalytic enzyme activities and is a rapidly emerging paradigm in medicine [1]. It combines benefits of high specificity in substrate recognition with signal amplification related to multiple catalytic cycles to produce specific and sensitive disease detection. Such amplification is particularly important in Magnetic Resonance Imaging (MRI), which suffers from intrinsic low sensitivity. Many pathologies are characterized by abnormal enzymatic activities, and the last two decades have seen very important chemistry efforts towards the development of chemical probes allowing for molecular imaging detection of a large variety of enzymes [2,3,4,5,6,7,8]. Given its high resolution, unlimited tissue penetration and non-invasiveness, MRI is particularly interesting for in vivo monitoring of enzymatic activities.

For instance, a whole network of protease activities is misregulated in multiple processes of cancer progression, such as tumor angiogenesis, invasion and metastasis [9]. Sensitive detection of proteases can be useful for diagnostic profiling, though so far clinical translation has been limited [10]. Key proteases in this context involve caspases, cathepsin B, urokinase and matrix metalloproteinases. Caspases are important as they lead to cell death by apoptosis, while tumors must escape apoptosis in order to progress towards malignancy. Urokinase, also known as urokinase plasminogen activator (uPA), is a serine protease that controls cancer invasion and metastasis. Elevated levels of uPA predict poor disease outcome in breast cancer. Binding of uPA to its receptor activates the enzyme that subsequently catalyzes the activation of other proteases, leading to degradation of the extracellular matrix.

Non-specific gadolinium(III) (Gd^3+^) complexes have been used for decades now in clinical MRI to improve image contrast by enhancing the relaxation rate of water protons in tissues [11]. The relaxation efficacy (relaxivity) of Gd^3+^ chelates is related to their structural and dynamic properties, such as the number of inner sphere water molecules (hydration number, *q*), their exchange rate with bulk water (*k*_ex_), or rotational dynamics (rotational correlation time, *τ*_R_). More recently, chemical exchange saturation transfer (CEST) appeared as another mechanism to create MRI contrast based on the exchange between labile protons of the imaging probe and water. Thanks to proton exchange, the selective saturation of these labile protons will also impact the water proton signal, and this signal decrease can be exploited to create MRI contrast [12]. The use of paramagnetic complexes as CEST probes is beneficial, since large paramagnetic shifts of the labile protons make selective saturation easier and allow for the exploitation of higher exchange rates, leading to more important PARACEST effects [13].

A great number of enzyme-responsive T_1_ and CEST probes have been proposed in the last two decades [2,3,4,14,15]. Some very recent studies report on hyperpolarized probes dedicated to enzymatic detection [6,8]. Gadolinium-free probes are also explored [7]. The T_1_ and CEST MRI signal changes induced by enzymatic activity can rely on different molecular strategies, including oligomerization or polymerization of the probe, disassembly of nanoparticles, modulation of the hydration number, solubility changes, relaxivity enhancement via protein binding or the formation of CEST-active chemical functions. In many examples, water coordination to Gd^3+^ is modulated by enzymatic transformation to result in a relaxivity change. For instance, in a recent innovative approach by Meade et al. to detect β-galactosidase, the Gd^3+^ coordination sphere was saturated by the coordination of an intramolecular carboxylate group or an independent carbonate to create a low-relaxivity complex. After enzymatic cleavage of the substrate and the concomitant decomposition of a self-immolative linker, this coordination could be impacted, resulting in a relaxivity increase, which could be used for in vivo detection of β-galactosidase [16]. Pagel and his group reported several successful enzyme-responsive probes based on PARACEST lanthanide as well as DIACEST agents [3,14,17,18]. In an early example for the detection of caspase-3, the substrate DEVD (Asp-Glu-Val-Asp), was conjugated to one sidearm of DOTA via an amide function. The amide proton of DEVD-(Tm-DOTA) showed a PARACEST effect at −51 ppm. Enzymatic cleavage by caspase-3 liberated a free amine group with a PARACEST effect at +8 ppm, allowing for enzyme detection [19]. More recently, uPA activity was assessed in a concentration-independent manner in vitro and in vivo by using two PARACEST agents [20] or a diamagnetic probe presenting two CEST signals [21]. Overall, there is an increasing number of enzymatic MR imaging agents attaining the preclinical stage of validation; however, there is still need for structural optimization based on a better understanding of their functioning mechanisms.

We have been long involved in the design of enzymatically activated imaging probes based on lanthanide complexes with detection capabilities in T_1_ or CEST MRI combined with optical detection [22,23,24]. Several systems have been developed with self-immolative linkers between the MRI-reporter lanthanide complex and the enzyme-specific substrate. A great advantage of self-immolative linkers is that the substrate cleavage site is remote from the metal chelate facilitating enzyme recognition; however, synthetic difficulties and the potential risk associated with the release of toxic side-products upon linker degradation might represent drawbacks [25].

**Scheme 1:**
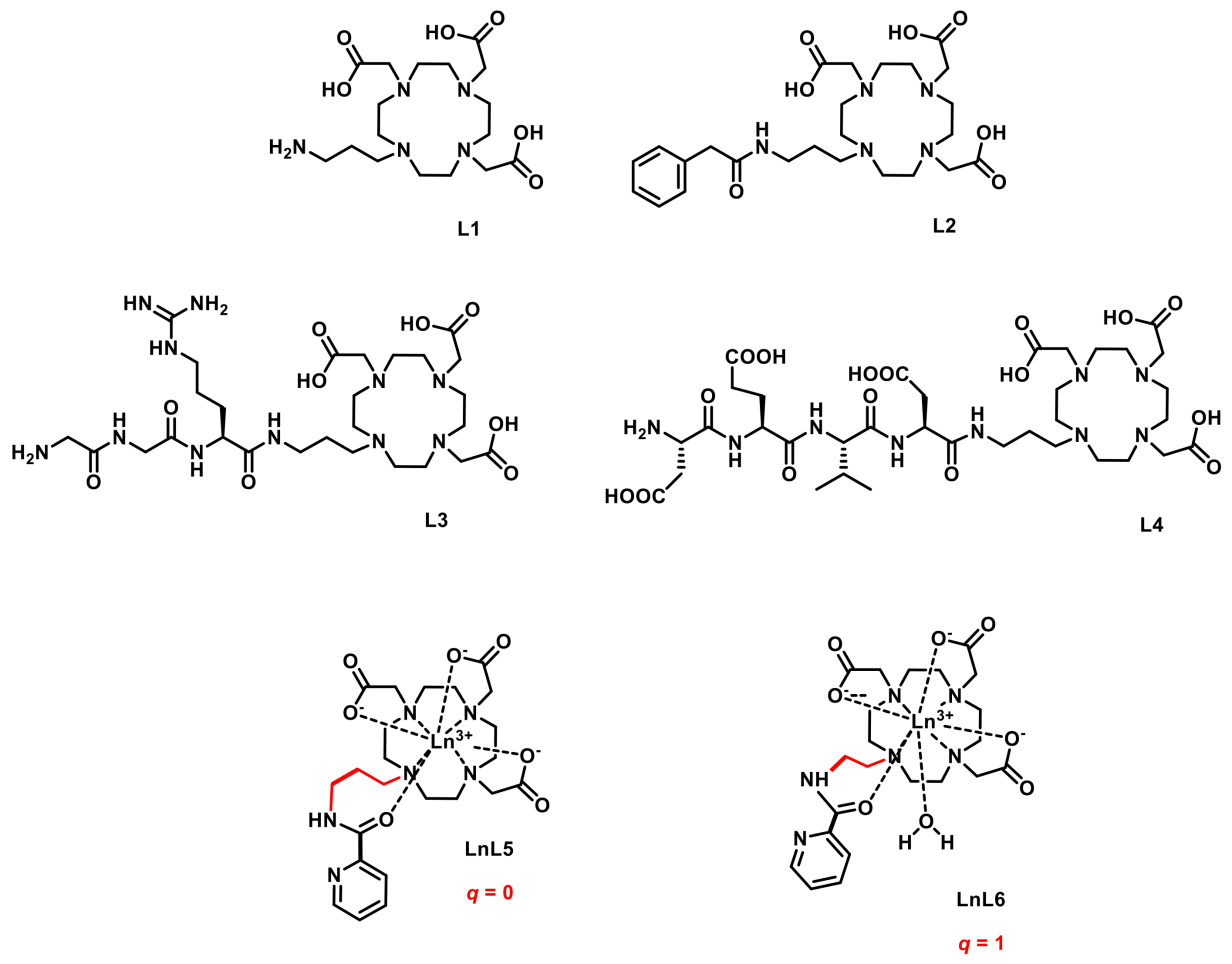
Structure of the ligands L1, L2, L3 and L4, and coordination modes for the LnL5 and LnL6 complexes [27] discussed in the text.

Here we report another initiative towards enzymatic probes based on a simpler molecular design with combined detection potential in T_1_ and PARACEST MRI using the same ligand complexed to different lanthanide(III) (Ln^3+^) ions. The complexes LnL3 and LnL4 bear the peptide substrates of urokinase and caspase-3, respectively, conjugated to the LnDO3A chelate via a simple propylene linker (Scheme 1). GGR (Gly-Gly-Arg) and DEVD (Asp-Glu-Val-Asp) are well-identified substrates of urokinase and caspase-3, respectively, which are efficiently and selectively cleaved by the enzymes at the C-terminus of the sequences and have been used in different imaging agents for detecting urokinase [20,21] or caspase-3 activity [19]. We have also synthesized LnL2 bearing the substrate of penicillin amidase, which does not have a particular biological interest but represents an analogue with a simple amide substituent. Phenylacetamide derivatives, which is the substrate for cleavage by the protease penicillin-G-amidase, are commonly used as model systems in the development of enzymatically activated probes, such as FRET-based agents [26].

Our design strategy followed on previous work by Congreve et al., which reported non-hydration of the propylene amide derivative complex EuL5, in sharp contrast to the mono-hydration observed for the ethylene-bridged analogue EuL6 [27] (Scheme 1). In EuL5, the amide oxygen is coordinated to the lanthanide ion, and the steric crowding induced by the eight-membered chelate ring prevents coordination of an additional water molecule; the overall coordination number (CN) is eight. We hypothesized thus that the Gd^3+^ complexes of ligands L2, L3 and L4 would also be non-hydrated, with a correspondingly low relaxivity. Nevertheless, the metal coordination of this amide function can be expected to be relatively weak, given the large chelate ring size. In solution, this could result in an equilibrium between the coordinated and non-coordinated state, which, even if strongly shifted towards the former, could ensure a certain proportion of the molecule recognizable by the enzyme, and thus amide coordination would not hinder enzymatic cleavage. Following the enzymatic reaction, we expect to recover the amine derivative complex GdL1, which should have two inner sphere water molecules and thus a higher relaxivity. If the coordinated metal ion is a paramagnetic lanthanide other than Gd^3+^, one can expect to observe a PARACEST signal for the amide protons of LnL2, LnL3 and LnL4, while LnL1 might generate a PARACEST effect related to its amine function.

We present here the synthesis of the ligands and the characterization of the GdL1, GdL2, GdL3 and GdL4 complexes with respect to their hydration state and relaxation properties. The corresponding Nd^3+^, Eu^3+^ and Yb^3+^ analogues have been investigated for their PARACEST behavior. Finally, enzymatic recognition was assessed for GdL3 and GdL4. Although these probes are not revealed to be useful for enzymatic detection, the present studies bring insight to the complexity of the relationships between the chemical structure of the sidechains in DO3A derivatives and the hydration state or MRI properties of the corresponding lanthanide complexes.

## 2. Results and Discussion

### 2.1. Syntheses of the Ligands and Complexes

**Scheme 2:**
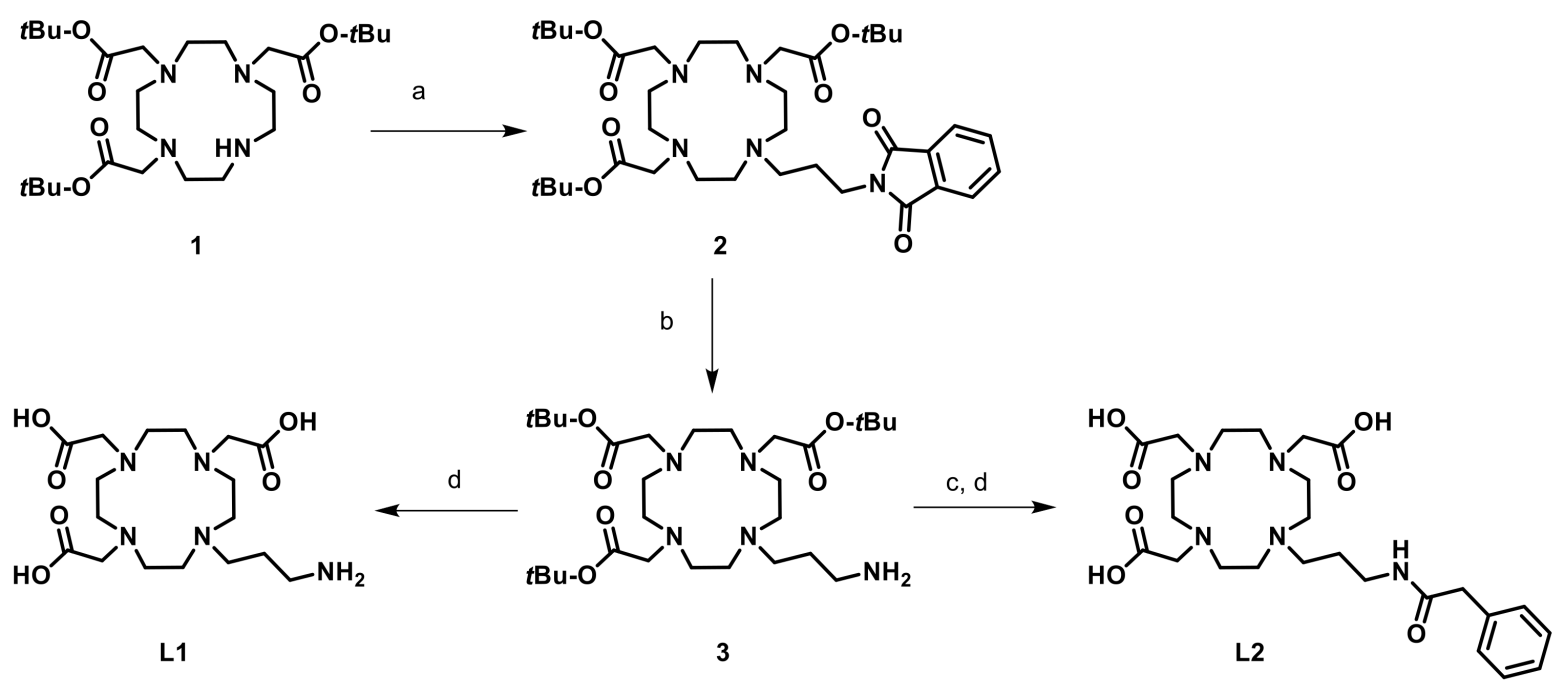
Synthesis of L1 and L2. The reagents and conditions were as follows: (**a**) *N*-(3-bromopropyl)phtalimide, K_2_CO_3_, NaI, CH_3_CN, reflux, 3 d, 70%; (**b**) NH_2_NH_2_·H_2_O, 90 °C, 30 min 60%; (**c**) phenylacetyl chloride, NEt_3_, THF, 1 h at 0 °C and 2 h at RT, 97%; (**d**) TFA, CH_2_Cl_2_, RT, 18 h, L1: 55%, L2: 80%.

The ligands L1 and L2 were prepared from the commercial tri-tert-butyl 2,2′,2″-(1,4,7,10-tetraazacyclododecane-1,4,7-triyl)triacetate (DO3A-tBu) in three and four steps, respectively (Scheme 2). The functionalized cyclen derivative 2 was obtained by *N*-alkylation of DO3A-tBu with *N*-(3-bromopropyl)phtalimide. Then, the primary amine 3 was obtained after removal of the phtalimide protecting group via an Ing-Manske procedure. The cleavage of the tert-butyl esters was carried out in acidic conditions using trifluoroacetic acid (TFA) in dichloromethane to afford ligand L1 with an overall yield of 23%. Ligand L2 was also prepared starting from compound 3 in two steps. A reaction of acylation with phenylacetyl chloride, followed by the deprotection of the protecting groups, in the same acidic conditions, produced ligand L2 with an overall yield of 32%.

**Scheme 3:**
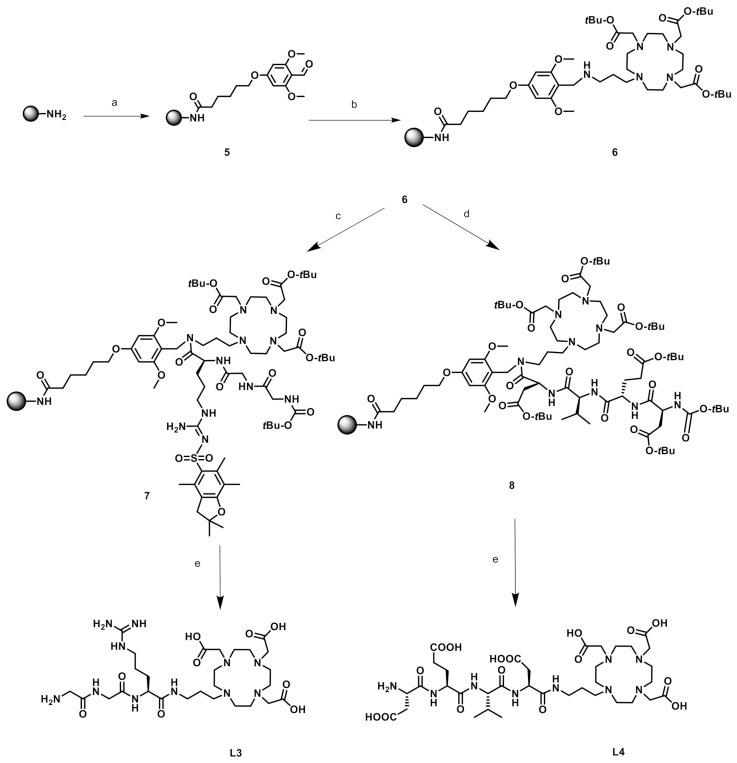
Synthesis of **L3** and **L4**. The reagents and conditions were as follows: (**a**) BAL linker, HATU/DIEA, DMF, RT, 18 h (**b**) **3**, NaBH_3_CN, DMF/MeOH/CH_3_COOH, RT, 18 h; (**c**) (i) Fmoc-Arg(Pbf)-OH, HATU/DIEA, DMF, RT, 18 h (ii) DMF/piperidine, RT, 3 × 3 min (iii) Fmoc-Gly-OH, HATU/DIEA, DMF, RT, 18 h (iv) DMF/piperidine, RT, 3 × 3 min (v) Boc-Gly-OH, HATU/DIEA, DMF, RT, 18 h; (**d**) (i) Fmoc-Asp(OtBu)-OH, Oxyma Pure/DIC, DMF, RT, 18 h (ii) DMF/piperidine RT, 3 × 3 min (iii) Fmoc-Val-OH, Oxyma Pure/DIC, DMF, RT, 18 h (iv) DMF/piperidine, RT, 3 × 3 min (v) Fmoc-Glu(OtBu)-OH, Oxyma Pure/DIC DMF, RT, 18 h (vi) DMF/piperidine, RT, 3 × 3 min (vii) Boc-Asp(OtBu)-OH, Oxyma Pure/DIC DMF, RT, 18 h; (**e**) TFA/H_2_O/TIPS, RT, 18 h.

Ligands L3 and L4 are composed of a sequence of three amino acids (GGR) and four amino acids (DEVD), respectively, linked to a macrocyclic part via a propyl-amide linker. Pagel et al. have developed several methods for incorporating an amine-derivatized DOTA at the C-terminus of such peptides. In an early work [28], a side chain-protected peptide was pre-assembled by solid phase peptide synthesis (SPPS) and then coupled in solution to the amino-DOTA, but such a fragment coupling approach is often associated with epimerization of the C-terminal amino acid residue [29]. To circumvent this issue, a functionalized DOTA derivative can be grafted on a solid support, and then the peptide sequence can be elongated through SPPS [30]. If this constitutes a more generally applicable method, it, however, necessitates complex synthesis of the functionalized macrocycle and requires several non-trivial solid-supported steps. In our case, as shown in Scheme 3, we chose to introduce, first, the backbone amide-linker 5-(4-formyl-3,5-dimethoxyphenoxy)valeric acid (BAL) on an aminomethylated polystyrene resin to get **5**. This spacer presents the advantage of being labile in acidic conditions and therefore will be removed during the final step of deprotection. The aldehyde function of the BAL spacer is also an easy way to link compound **3** to the solid support through a reductive amination reaction. The derivative **6** obtained is common to the ligands L3 and L4 and is the starting point of peptides GGR and DEVD building, using standard Fmoc-based solid phase synthesis to afford the **7** and **8** derivatives. The DEVD sequence of L4 presents an N-terminal aspartic acid residue that could undergo aspartimide formation under the prolonged coupling conditions we use. Such cyclization is base-catalyzed, so we decided to prevent this potential side reaction by changing the coupling conditions: 1-[bis(dimethylamino)methylene]-1H-1,2,3-triazolo[4,5-b]pyridinium 3-oxide hexafluorophosphate/*N*,*N*-diisopropylethylamine (HATU/DIEA), used for L3, has been replaced by Oxyma Pure (ethyl cyanohydroxyiminoacetate)/*N*,*N*′-diisopropylcarbodiimide (DIC), without using any additional base. The simulaneous cleavage of all the protecting groups and the BAL linker by reaction with TFA in the presence of triisopropylsilane (TIPS) as a carbocation scavenger, followed by reverse phase chromatography purification, produced ligands L3 and L4 with 80% and 76% overall yield, respectively.

### 2.2. Relaxometric Characterization of the Gd^3+^ Complexes

**Figure 1:**
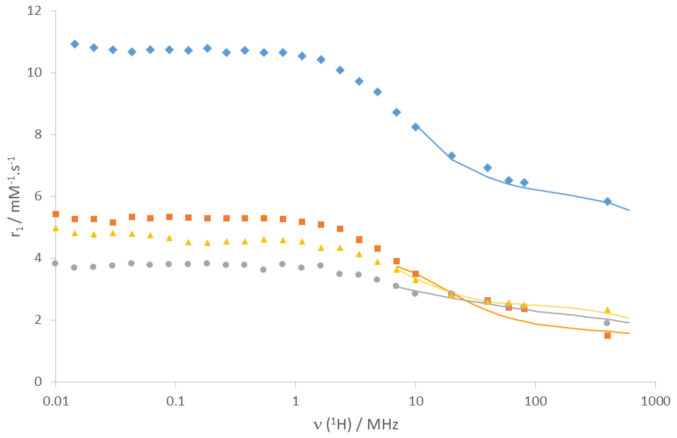
^1^H Nuclear Magnetic Relaxation Dispersion (NMRD) profiles of GdL1 (◆), GdL2 (■), GdL3 (●) and GdL4 (▲) at 37 °C. The lines represent the best fit to the SBM equations.

In order to assess the potential difference of relaxivity between the model complexes before (GdL2, GdL3, GdL4) and after (GdL1) enzymatic cleavage, ^1^H Nuclear Magnetic Relaxation Dispersion NMRD profiles have been recorded between 10 kHz and 400 MHz at different temperatures and pH 7. The results are presented in Figure 1 and Figures S1–S4. For all the complexes, the relaxivity decreases with increasing temperature (Figures S1–S4), which is characteristic of low molecular weight, fast tumbling systems. As compared with GdL2–4, a higher relaxivity (ca. 160% at 37 °C and 20 MHz) is observed for GdL1, which mimics the complex after enzymatic cleavage. Surprisingly, the relaxivity of GdL2 is remarkably higher than that reported for GdL5 (5.17 mM^−1^·s^−1^ vs. ca. 3 mM^−1^·s^−1^ at 10 MHz, 25 °C) [27], although they differ only by having a pyridine in GdL5 in place of the benzyl group in GdL2. The low relaxivity of GdL5 was explained by the absence of any water molecules in the first coordination sphere of Gd^3+^, as determined by luminescence lifetime measurements on the corresponding Eu^3+^ complex. Non-hydration was ascribed to the steric hindrance of the propyl linker as the same complex with an ethyl linker (LnL6, Scheme 1) had one water molecule in the first coordination sphere, and the corresponding Gd^3+^ complex had a relaxivity of 4.5 mM^−1^·s^−1^ in the same conditions [27].

In order to better characterize the coordination sphere of the complexes and to determine the number of water molecules directly coordinated to Ln^3+^, luminescence lifetime measurements have been performed on the EuL1–4 analogues in H_2_O and D_2_O. All the data could be fitted by monoexponential decay functions indicating that one species is present in solution. When the deactivation of Ln^3+^ is occurring mainly through the X-H oscillator vibrations (X = O, C, N), the following empirical formulae ((1), (2)) can be used to estimate the number of water molecules directly coordinated to the Eu^3+^ [31]:

(1) $$q_{\mathrm{Eu}}=1.11\left(\tau_{H2O}^{-1}-\tau_{D2O}^{-1}-k_{\mathrm{XH}}\right)$$

(2) $$k_{\mathrm{XH}}=0.30+0.45n_{\mathrm{OH}}+0.99n_{\mathrm{NH}}+0.075n_{O=\mathrm{CNH}}$$

where τ represents the luminescence lifetimes, and n_OH_, n_NH_, and n_O=CNH_ are respectively the number of alcoolic, amine and amide oscillators in the first coordination sphere of Eu^3+^ (in the case of the amide, the coordination is through the oxygen).

The results are presented Table 1. These data, interpreted together with the relaxivities measured for the corresponding Gd^3+^ chelates (Table 1), allow us to draw the following conclusions.

(a) As expected, EuL1 has the highest number of coordinated water molecules of the whole series (*q* ≈ 2), with a correspondingly high relaxivity for the Gd^3+^ analogue.
(b) EuL2 has one coordinated water, implying that the coordination of the amide function still leaves space for one water molecule in the inner sphere. Despite their similar structure, this is in sharp contrast to LnL5, which has no inner-sphere water. Likely due to its higher flexibility around the methylene group, the benzyl moiety of LnL2 does not seem to hinder the access of the water molecule to Ln^3+^ as much as the pyridine. This difference highlights the difficulty of predicting the coordination mode, even in structurally analogous complexes. The hydration number *q* = 1 of GdL2 is in full accordance with its relaxivity, 70% higher than that of GdL5 [27].
(c) EuL4 revealed to be non-hydrated, i.e., in addition to the amide coordination, either a carboxylate function from the peptide side chain (aspartate or glutamate) completes the coordination sphere to CN = 9, or the peptide is too “bulky” and prevents water access to Gd^3+^ (CN = 8). The relaxivity of GdL4 is remarkably high for a non-hydrated complex. Indeed, in the absence of hydration water, the relaxivity should be purely of outer-sphere origin, governed by the diffusion of bulk water molecules in the vicinity of the Gd^3+^. A rough estimation of an outer-sphere relaxivity gives a value of ca. 2.7 mM^−1^·s^−1^ at 20 MHz, 25 °C. For the outer-sphere mechanism, relaxivity is mainly determined by diffusion (of water molecules and the complex), and the distance of closest approach between water protons and Gd^3+^. Although the distance can vary depending on the geometry of the complex, this cannot plausibly account for such a high relaxivity. Much more likely, a second-sphere relaxivity mechanism operates due to the numerous carboxylate and amide functions of the peptid side-chain, which can retain water molecules in the vicinity of Gd^3+^ by hydrogen bonds.
(d) For LnL3, the interpretation of the luminescence lifetimes and the estimation of *q* are more complex. In a first consideration, one can hypothesize a similar coordination mode to that of LnL2, involving the coordination of the macrocycle amines, the three macrocycle carboxylates and the amide to the Ln^3+^ ion. In this case, similarly to EuL2, Equation 1 indicates monohydration (*q* = 0.7(3)). However, this is not coherent with the relaxivities, which, for small molecular complexes like GdL2 and GdL3, are mainly dictated by rotation and thus molecular size. Given the bigger size of GdL3, if both GdL2 and GdL3 were monohydrated, GdL3 should have superior relaxivity. However, it is even lower than the relaxivity of the non-hydrated GdL4, suggesting that the number of water molecules coordinated could be rather close to 0. In LnL3, the amine functions of the peptide side chain can also potentially coordinate to the metal, which should then be taken into account in Equation 1. Considering the extra coordination of an amine in Equation 1, *q* = −0.4 is estimated for EuL3. Given the error on this value, the extra nitrogen coordination to Ln^3+^ leads to a *q*-value close to 0. (Though less likely due to steric reasons, an amide of the peptide chain might also coordinate; in this case, the estimated *q* would be 0.6). Overall, the combined analysis of the relaxivities and the luminescence lifetimes suggests that no water molecule is present in the first coordination sphere of LnL3, and the relaxivity of GdL3 is governed by a second sphere effect, similarly to GdL4.

In order to gain insight into the parameters governing relaxivity, the NMRD profiles of the four complexes have been fitted using the Solomon-Bloembergen and Morgan (SBM) theory. If we are not interested in electronic parameters, the fit can be restricted to frequencies higher than 6 MHz. The parameters obtained from the fit are presented in Table 2 (see Table S1 for the full set). Several parameters have been fixed to common values, such as the relative diffusion coefficient D_GdH_^298^ = 2.6 × 10^−9^ m^2^·s^−1^, the closest approach between the Gd^3+^ ion and the proton a_GdH_ = 3.5 Å, and the activation energy of the modulation of the zero field splitting E_V_ = 1 kJ·mol^−1^. The rotational correlation time, *τ*_R_, and its activation energy, E_r_; the activation energy of the relative diffusion coefficient, E_DGdH_; and the electronic parameters, i.e., the mean square of the zero field splitting Δ^2^ and the correlation time for the modulation of the zero field splitting *τ*_V_^298^, have been fitted, but these electronic parameters should not be over-interpreted (Table S1).

In the case of GdL1, *q* was fixed to 2, and the water exchange rate was set to the values of GdDO3A: *k*_ex_^298^ = 11 × 10^6^ s^−1^ and ΔH^≠^ = 33.6 kJ·mol^−1^ [32]. For GdL2, *q* was fixed to 1, and the water exchange rate and activation enthalpy were set to the values determined for GdL6, based on the analogy of the coordination sphere [27]. For both systems, a classical value of the Gd-water proton distance, 3.1 Å, was used. The fit yielded *τ*_R_ = 95 ps and 55 ps for GdL1 and GdL2, respectively. The value for GdL2 is similar to that reported for GdL6 (60 ps) [27]. The longer rotational correlation time of GdL1 suggests a less compact structure for the complex, in accordance with the non-coordinating and protonated propylamine arm.

To analyse the NMRD profiles of GdL3, we first proceeded using *q* = 1 and the same *k*_ex_^298^ and ΔH^≠^ as for GdL2. This fit yielded *τ*_R_ = 49 ps (see Table S1). This value even seems overestimated, as illustrated in Figure S3, since the experimental relaxivities at a high field, where *τ*_R_ is best determined, are well below the fitted curve. Such low *τ*_R_ value is not reasonable, given the larger size of GdL3 vs. GdL2. Therefore, as explained above, we assume that the coordination sphere of Gd^3+^ is completed by an extra amine from the arginine side-chain, and in the analysis of the NMRD curves, we set *q* to 0 and took into account a second-sphere contribution to the relaxivity. To describe this mechanism, the Gd-H distance was fixed to 3.8 Å, and the exchange rate of the second sphere water was assumed to be very fast, *k*_ex_^298^ = 2 × 10^9^ s^−1^ [33]. In reality, the exchange of the second sphere water is too fast to have any impact on the fitting. The best fit was obtained with assuming one second-sphere water molecule (*q^SS^* = 1), resulting in a realistic value for the rotational correlation time, *τ*_R_ = 160 ps (Figure S3). When assuming two second-sphere water molecules (*q^SS^* = 2), we obtained *τ*_R_ = 88 ps, which again seems too short for this molecular size; moreover, the agreement between the fit and the experimental data is less good (the sum of the least squares is 1.3 × 10^−1^ for *q^SS^* = 2 vs. 6.2 × 10^−2^ for *q^SS^* = 1). We can therefore safely conclude that the relaxivity of GdL3 can be well described by the presence of one second-sphere water molecule with a Gd-H distance of 3.8 Å.

Likewise, for GdL4 we have assumed one second-sphere water molecule to account for the relaxivity. A *τ*_R_ value of 260 ps is obtained in the fit, which is reasonable for the size of the complex.

In conclusion, the comparison between these complexes shows again that small structural variations in the ligand can induce important differences in the first coordination sphere of the Ln^3+^. The peptidic complexes likely provide extra coordinating functions to complete the coordination sphere of Ln^3+^, although one cannot fully exclude that they do not coordinate, but their bulkiness prevents water access to the metal. The lack of inner sphere water is partially compensated in these highly hydrophilic complexes by second-sphere water molecules, which maintain relatively high relaxivities. Overall, the assumed enzymatic transformation of GdL3 and GdL4 to GdL1 would still lead to a sizeable increase in relaxivity (ca. 160% from the relaxivity measurements performed at 37 °C and 20 MHz).

### 2.3. ParaCEST Properties of Eu^3+^, Yb^3+^ and Nd^3+^ Complexes

**Figure 2:**
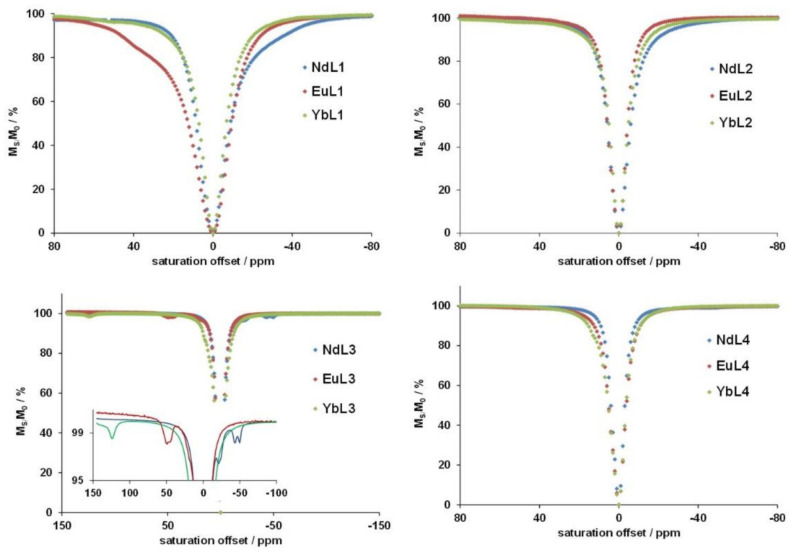
CEST spectra of LnL1, LnL2, LnL3 and LnL4 complexes recorded at 400 MHz, pH 7.4, 298 K, using a pre-saturation pulse of 3 s at 25 µT (10 mW). The concentration of the samples were: [EuL1] = 82 mM, [EuL2] = 54 mM, [EuL3] = 18 mM, [EuL4] = 18 mM, [NdL1] = 90 mM, [NdL2] = 83 mM, [NdL3] = 18 mM, [NdL4] = 17 mM, [YbL1] = 90 mM, [YbL2] = 82 mM, [YbL3] = 16 mM, [YbL4] = 19 mM.

Amine and amide functions are commonly reported to produce PARACEST effects in complexes of paramagnetic lanthanide ions. We have recorded PARACEST spectra for the Nd^3+^, Eu^3+^ and Yb^3+^ chelates of the four ligands, L1, L2, L3 and L4, at 25 °C, in a spectral window of 300 ppm (from −150 to +150 ppm; Figure 2). These three metal ions are representative of the beginning, the middle and the end of the lanthanide series and are often used to generate PARACEST effects. The PARACEST spectra are shown at pH 7.4 for all systems; in the pH range 6–8, apart from slight changes in the CEST intensities, no significant variation has been detected in the spectra for any of the complexes.

All the CEST effects observed are relatively weak. For the amine derivatives, EuL1 and NdL1 have a stronger effect at ~+40 ppm and ~−40 ppm, respectively, while YbL1 shows minor (~2%) CEST at 50 ppm. In a previous report, the Eu^3+^ and Yb^3+^ complexes formed with the ethylamine analogue of L1 were shown to produce CEST (the effects were considerably more important, but spectra were recorded in a 1:9 H_2_O:D_2_O solvent mixture) [34]. Based on a strong decrease in the amine protonation constant from the ethylamine derivative ligand (pKa = 10.51) to its Gd^3+^ complex (pKa = 5.83), the authors concluded that the amine function was coordinated, in a deprotonated form, to the metal ion, and this proximity of the amine protons to the paramagnetic Eu^3+^ or Yb^3+^ was important for the generation of the PARACEST effect. In contrast, for LnL1 complexes, the bishydrated nature of the metal ion clearly excludes amine coordination. In this case, the amine protons remain more distant, resulting in less important CEST effects.

The CEST spectrum of NdL2 shows a weak, assymetric broadening of the water peak, while the two other LnL2 complexes remain silent. The situation is similar for the LnL4 analogues; here, the Yb^3+^ complex has a low intensity peak at around 13 ppm. Obviously the single amide proton, even in the proximity of the lanthanide ion, is not sufficient to produce a more important CEST effect. Indeed, most Ln^3+^ amide complexes reported to generate significant CEST possess four equivalent amide protons [35,36].

The case of LnL3 chelates is different, though, as all three complexes present several weak CESTs at different frequencies. These multiplied CEST signals can be attributed (i) to different LnL3 isomers in solution, (ii) to the presence of the guanidinium protons in the side-chain, which, in addition to the coordinating amide, can also result in a CEST effect—or likely both (i) and (ii). DOTA-type Ln^3+^ complexes can form Square Anti Prismatic (SAP) and Twisted Square Anti Prismatic (TSAP) isomers [37]. The ^1^H NMR spectra of YbL3 recorded at pH 6.1 and 8.2 (Figure S5) show a large number of peaks with varying intensities as a function of pH, indicating that the major SAP isomer at pH 6.1 becomes minor at pH 8.2. Indeed, according to literature data on DOTA-type complexes, the axial protons of the macrocycle are the most shifted, and they typically have larger paramagnetic shifts in the SAP form [37].

Overall, the only exploitable PARACEST effects are observed for LnL1 (Ln = Eu, Nd), which could provide a “switch on” signal upon enzymatic conversion of the corresponding LnL3 or LnL4 chelates.

### 2.4. Enzymatic Cleavage

**Figure 3:**
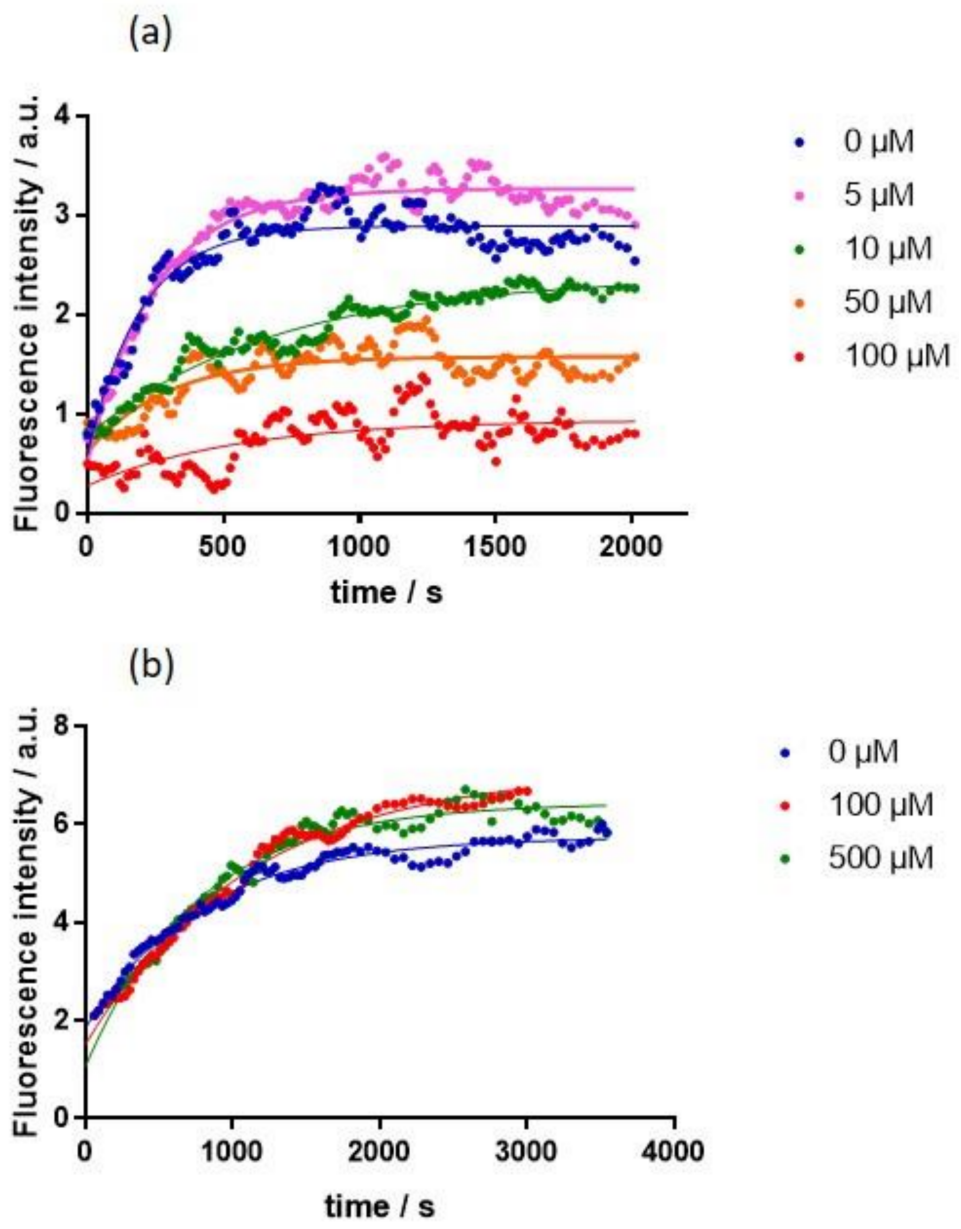
Fluorescence competition experiments of enzymatic cleavage. (**a**) Enzymatic transformation of 0.5 μM of *N*-Cbz-GGR-AMC by urokinase (0.5 U) in the presence of increasing GdL3 concentrations (0–100 μM). pH 7.5, 37 °C, BSA 7 mg/mL; λ_ex_ = 365 nm, λ_em_ = 440 nm. (**b**) Enzymatic transformation of 0.7 μM of *N*-acetyl-DEVD-AFC by caspase-3 (0.005 U) in the presence of increasing GdL4 concentrations (0–500 μM). pH 7.4, 37 °C; λ_ex_ = 380 nm, λ_em_ = 460 nm. Lines are drawn to show the tendency.

The potential transformation of GdL3 and GdL4 by their corresponding enzymes, respectively urokinase and caspase-3, have been assessed in vitro by relaxivity measurements. The relaxivity of GdL3 (1.2 mM) was monitored at 37 °C and pH 7.5 in the presence of 500 U of urokinase (in 0.5 mL), 50 mM phosphate buffer and 7 mg/mL Bovin Serum Albumin (BSA). The initial relaxivity measured in this solution at 20 MHz corresponds well to *r*_1_ determined in pure water, excluding BSA binding of the complex. Unfortunately, no relaxivity change was detectable in the sample even after 24 h. We then tried a fluorescent competition study by using the commercial dye *N*-Cbz-GGR-AMC (AMC = 7-amido-methylcoumarine), which bears the same peptide substrate of urokinase as GdL3. Following enzymatic cleavage of the substrate, the emission intensity of AMC strongly increases. The presence of increasing amounts (0–140 equivalents) of GdL3 strongly affects the time-dependent evolution of the fluorescence emission of *N*-Cbz-GGR-AMC, implying that there is an interaction between the enzyme and GdL3 (Figure 3). GdL3 inhibits enzyme activity but does not undergo enzymatic cleavage. As discussed above, in addition to the amide function of the linker, the coordination of an arginine amine from the side-chain is also suspected. The lack of enzymatic conversion suggests that the binding of these functions to the metal is relatively strong and prevents the access of the enzyme to the substrate.

In a similar way, no enzymatic conversion could be observed for 24 h by relaxivity measurements in a GdL4 (0.94 mM) solution in the presence of 6.69 × 10^−3^ U of caspase-3 (corresponding to ~60 nM), 50 mM HEPES buffer (pH 7.4), 0.1 M NaCl and 10 mM DTT (1,4-dithiothreitol) as the reducing agent, at 37 °C. In this case, the fluorescent competition assay showed practically no impact of up to 700 equivalents of GdL4 on the enzymatic transformation of the dye *N*-acetyl-DEVD-AFC (AFC = 7-amido-4-trifluoromethylcoumarine; Figure 3). Again, the combined relaxivity (GdL4) and luminescence (EuL4) data suggest potential coordination of one carboxylate group of the peptide side chain to the metal ion. This hypothesis seems to be supported also by the fact that the DEVD substrate is not recognized by the enzyme.

## 3. Materials and Methods

### 3.1. General Information

Solvents and reagents came from different suppliers such as Sigma-Aldrich (Saint Louis, MS, USA), Alfa-Aesar (Haverhill, MA, USA), TCI Europe (Anvers, Belgium), Fisher Scientific (Waltham, MA, USA), or CheMatech (Dijon, France). Hydrazine is highly toxic and potentially explosive and must be handled very carefully. Polypropylene syringes fitted with polypropylene frits were obtained from Torviq (Niles, MI, USA) and were equipped with PTFE stopcock bought from Biotage. Caspase-3 from E. Coli was purchased from Sigma (ref. C1224). The lyophilized powder contains 6.69 units/mg of protein.

The reactions were monitored by thin layer chromatography (TLC) on silica gel F254 plates. The plates were revealed depending on the conditions required by the use of an ultraviolet lamp (254 nm) or by using chemical dyes such as potassium permanganate solution (KMnO_4_), ninhydrin or Dragendorff’s solution. Flash chromatographies were performed using Interchim flash chromatography Spot II device.

HPLC analyses were carried out on a LaChromElite system equipped with a Hitachi L-2130 pump, a Hitachi L-2455 diode array detector and a Hitachi L-2200 auto sampler. Chromolith High Resolution RP-18e (150 Å, 10 × 4.6 mm, 3 mL/min flow rate) columns were used for analysis.

^1^H and ^13^C NMR spectra were recorded on a Bruker Avance Spectrometer using a 5 mm BBFO probe at 599.903 and 150.860 MHz for ^1^H and ^13^C, respectively. High Resolution Mass Spectrometry (HRMS) analyses were performed on a maXisTM ultra-high-resolution Q-TOF mass spectrometer (Bruker Daltonics, Bremen, Germany) through electrospray ionization using the positive mode. The structures of compounds for the NMR attribution are presented in the Supporting Information (Figures S6–S12).

### 3.2. Synthesis

The synthesis of compounds **2**, **3** and L1 is described in the Supporting Information.

**2,2′,2″-(10-(3-(2-Phenylacetamido)propyl)-1,4,7,10-tetraazacyclododecane-1,4,7-triyl) triacetic acid: L2**

To a solution of compound **3** (630 mg, 1.10 mmol) in tetrahydrofuran (10 mL) at 0 °C, was added successively triethylamine (0.35 mL, 2.8 mmol) and phenylacetylchloride (0.26 mL, 1.98 mmol). The reaction mixture was stirred for 1h at 0 °C and 2 h at room temperature. The reaction was then quenched with 10 mL of a K_2_CO_3_ solution, and the tetrahydrofuran was evaporated under a vacuum. The product was then extracted (3 × 30 mL) with dichloromethane. The organic phase was collected, dried over sodium sulfate, and the solvent was removed under the vacuum to give 740 mg of yellow oil with 97% yield.

^1^H NMR: (CDCl_3_, 600 MHz, δ ppm) = 1.44 (9H, s, H29); 1.45 (18H, s, H27,31); 1.59 (2H, tt, ^3^J_H9–H8_ = ^3^J_H9–H10_ = 6.6 Hz, H9), 2.51 (2H, bs, H10); 2.58–2.73 (16H, m, 11,12,13,14,15,16,17,18); 3.15–3.29 (8H, m, H8,19,20,21); 3.53 (2H, s, H7); 7.12–7.34 (5H, m, H1,2,3,5,6).

^13^C NMR: (CDCl_3_, 150 MHz, δ ppm) = 25.6 (C9); 27.9 (C29); 28.2 (C27,31); 43.2 (C8); 43.3 (C7); 49.0(C12,17); 51.6 (C10); 52.2 (C16,13); 52.6 (C11,18); 53.6 (C14,15); 56.2 (C20); 56.6 (C19,21); 80.9 (C26,28,30); 127.0 (C1); 128.6 (C2,6); 129.5 (C3,5); 134.0 (C4); 170.6(C23,24); 170.8 (C22).

HRMS: calculated for C_37_H_64_N_5_O_7_ [M+H]^+^: *m*/*z* = 690.4800, found [M+H]^+^: *m*/*z* = 690.4803.

The yellow oil obtained (737 mg, 1.07 mmol) was dissolved in dichloromethane (20 mL), and trifluoroacetic acid (24 mL, 320 mmol) was added. The reaction mixture was stirred for 18 h at room temperature. The product was then evaporated under a vacuum. The resulting oil was dissolved in a small amount of dichloromethane, and cold diethyl ether was added. The precipitate obtained was solubilized in a small amount of water and purified by Flash chromatography on RPC18 phase to give L2 as a yellowish powder with a yield of 82%.

^1^H NMR: (D_2_O, 600 MHz, δ ppm) = 1.86 (2H, tt, ^3^J_H9–H8_ = ^3^J_H9–H10_ = 6.7 Hz, H9); 2.74–3.00 (8H, m, H12,13,16,17); 2.93 (2H, m, H10); 3.19 (2H, m, H8); 3.22 (4H, m, H11,18); 3.38 (4H, m, H14,15); 3.39 (4H, m, H19,21); 3.47 (2H, s, H7); 3.99 (2H, s, H20); 7.22–7.32 (5H, m, H1,2,3,5,6).

^13^C NMR: (D_2_O, 150 MHz, δ ppm) = 23.1 (C9); 36.3 (C8); 42.6 (C10); 48.0 (C14,15); 48.1 (C12,17); 48.3 (C13,16); 49.9 (C11,18); 52.1 (C20); 52.9 (C19,21); 55.3 (C7); 127.4 (C1); 128.9 (C2,6); 135.1 (C4); 174.1 (C25); 175.0 (C22,23,24).

HRMS: calculated for C_25_H_40_N_5_O_7_: *m*/*z* = 522.2922, found: *m*/*z* = 522.2925.

**(S)-2,2′,2″-(10-(3-(2-(2-(2-aminoacetamido)acetamido)-5-guanidinopentanamido)propyl)-1,4,7,10-tetraazacyclododecane-1,4,7-triyl)triacetic acid: L3**

Peptide elongation was performed manually on an aminomethylated polystyrene resin (0.056 mmol, 1.4 mmol/g), using standard Fmoc/tert-butyl chemistry with HATU/DIEA activation in DMF (2 equiv. carboxylic acid, 1.95 equiv. HATU, 4 equiv. DIEA, 0.5 M carboxylic acid concentration) for 18 h at RT. All solid-supported reactions were performed on polypropylene syringes fitted with polypropylene frits using rotation stirring. After coupling the BAL linker, DO3A-tBu-amine 3 was incorporated through reductive amination (1.1 equiv. 3, 5 equiv. NaBH_3_CN, DMF/MeOH/CH_3_COOH 45:45:10, RT, 18 h). The elongation was pursued by Fmoc-SPPS using Fmoc-Arg(Pbf)-OH, Fmoc-Gly-OH and Boc-Gly-OH. When needed, coupling reactions were repeated until total peptidic coupling, monitored through the Kaiser test, or the chloranyl test for the BAL secondary amine. After each Fmoc-amino acid coupling, the Fmoc protecting group was removed with a treatment by 20% piperidine in DMF (3 × 3 min). The Fmoc deprotection reactions were monitored by measuring the UV absorbance (301 nm) of the formed dibenzofluvene-piperidine adduct (ε = 7800 M^−1^·cm^−1^). Final deprotection and cleavage from the solid support were performed with TFA/H_2_O/TIS: 90/5/5 by vol for 2 h (10 mL). The crude peptide was precipitated in cold Et_2_O/petroleum ether: 1/1 by vol (100 mL), centrifugated, and washed twice with Et_2_O, followed by centrifugation; then, it was solubilized in deionized water and lyophilized. Purification was performed by reversed-phase flash chromatography using a linear water-acetonitrile gradient with 0.1% TFA. A white powder was obtained with an overall yield of 80%.

^1^H NMR: (D_2_O, 600 MHz, δ ppm) = 1.50 (2H, m, H7); 1.66 (2H, m, H6); 1.86 (2H, m, H12); 2.94 (4H, m, H15, 20); 3.01 (4H, m, H16, 19); 3.07 (2H, t, ^3^J = 6.9 Hz, H8); 3.17 (4H, m, H11,13); 3.29 (4H, m, H14, 21); 3.41 (4H, m, H17,18); 3.43 (4H, m, H22, 26); 3.77 (2H, s, H1); 3.90 (2H, s, H3); 4.09 (2H, s, H24); 4.10 (1H, m, H5).

^13^C NMR: (D_2_O, 150 MHz, δ ppm) = 22.9 (C12); 24.4 (C7); 28.0 (C6); 36.4 (C11); 40.4 (C1); 40.4 (C8); 42.3 (C3); 48.0 (C15, 20); 48.4 (C19, 16); 49.9 (C14, 21); 51.8 (C17, 18); 52.0 (C13); 52.9 (C22, 26); 53.8 (C5); 54.1 (C24); 156.7(C9); 167.7 (C2); 168.5 (C25); 171.2 (C4); 173.8 (C10); 174.3 (C23, 27).

HRMS: calculated for C_27_H_52_N_11_O_9_ [M+H]^+^: *m*/*z* = 674.3943, found [M+H]^+^: *m*/*z* = 674.3948.

Analytical HPLC: t_R_ = 1.70 min, gradient: 0 to 5% B in 5 min.

**2,2′,2″-(10-((6S,9S,12S,15S)-15-amino-16-carboxy-12-(2-carboxyethyl)-6-(carboxymethyl)-9-isopropyl-5,8,11,14-tetraoxo-4,7,10,13-tetraazahexadecyl)-1,4,7,10-tetraazacyclododecane-1,4,7-triyl)triacetic acid: L4**

The synthetic procedure has been adapted from that of L3 by replacing HATU/DIEA with Oxyma Pure/DIC (2 equiv. each). L4 was obtained as a white powder with an overall yield of 76%.

^1^H NMR: (D_2_O, 600 MHz, δ ppm) = 0.82 (3H, d, ^3^J = 6.8 Hz, H12); 0.83 (3H, d, ^3^J = 6.8 Hz, H13); 1.89 (2H, m, H20); 1.95 (2H, m, H6); 1.96 (1H, m, H11); 2.37 (2H, m, H7); 2.77 (2H, m, H2); 2.97 (2H, m, H16); 2.98 (2H, m, H19); 3.00 (4H, m, H23,28); 3.06 (4H, m, H24,27); 3.16 (2H, m, H21); 3.32 (4H, m, H22,29); 3.44 (4H, s, H30,34); 3.46 (4H, m, H25,26); 3.94 (1H, d, ^3^J = 7.5 Hz, H10); 4.13 (2H, s, H32); 4.28 (1H, t, ^3^J = 6.1 Hz, H15); 4.36 (1H, t, ^3^J = 7.1 Hz, H5); 4.52 (1H, t, ^3^J = 6.8 Hz, H3).

^13^C NMR: (D_2_O, 150 MHz, δ ppm) = 17.8 (C12); 18.3 (C13); 22.9 (C20); 26.1 (C6); 29.8 (C7); 29.9 (C11); 34.6 (C19); 35.3 (C2); 36.5 (C16); 48.0 (C23,28); 48.5 (C24,27); 49.4 (C15); 50.0 (C22,29); 50.3 (C3); 51.9 (C34,30); 52.1 (C21); 52.9 (C25,26); 53.2 (C5); 54.6 (C32); 59.8 (C10); 168.4 (C33); 168.5 (C18); 172.1 (C9); 172.4 (C4); 172.9 (C14); 173.0 (C17); 173.8 (C1); 174.3 (C31,35); 176.8 (C8).

HRMS: calculated for C_35_H_60_N_9_O_16_ [M+H]^+^: *m*/*z* = 862.4152, found [M+H]^+^: *m*/*z* = 862.4156.

Analytical HPLC: t_R_ = 3.56 min, gradient: 0 to 10% B in 5 min.

### 3.3. Sample Preparation

Solutions of Ln^3+^ were prepared in MILLIQ (ultrapure) distilled water, and their concentrations were determined by complexometric titration with a standardized EDTA solution (H_4_EDTA = ethylenediaminetetraacetic acid) using xylenol orange as an indicator. The purity of the ligands was assessed by adding an excess of Zn^2+^ followed by complexometric titration of the non-complexed Zn^2+^ in solution with EDTA. The different complexes were prepared by mixing equimolar quantities of cation and ligand solutions, which were stirred for 1h at room temperature. During complexation, the pH was monitored and maintained between pH = 4–5. After a 1h reaction, a xylenol orange test was performed to ensure total complexation of the metal. The lanthanide concentration of the prepared complexes was controlled either by ICP (Inductively Coupled Plasma) or BMS (Bulk Magnetic Susceptibility) techniques.

### 3.4. Relaxometric Measurements

Proton NMRD measurements were performed on a Stelar SMARTracer Fast Field Cycling relaxometer (0.01–10 MHz) and a Bruker WP80 NMR electromagnet adapted to variable field measurements (20–80 MHz) and controlled by a SMARTracer PC-NMR console. The temperature was monitored by a VTC91 temperature control unit and maintained by a gas flow. The temperature was determined by previous calibration with a Pt resistance temperature probe. 1/T_1_ longitudinal relaxation rates were determined for GdL1, GdL2, GdL3 and GdL4 under the following conditions: [GdL1] = 6.39 mM, pH 7.00, [GdL2] = 1.23 mM, pH 7.17, [GdL3] = 0.96 mM, pH 7.36, [GdL4] = 0.93 mM, pH 6.98 and different temperatures (298 K, 310 K, and 323 K).

### 3.5. Luminescence Lifetime Measurements

The luminescence lifetime measurements of the europium complexes were performed on an Agilent Cary Eclipse Fluorescence spectrophotometer by recording the decay of the emission intensity at 616 nm, following the direct excitation of the lanthanide ion at 396 nm. Measurements were performed for both H_2_O and D_2_O solutions at a pH/pD of 7.4 and a concentration of 1 mM. The settings were as follows: gate time: 0.05 ms; delay time: 0.1 ms; total decay time: 10 ms; 100 cycles.

At least three decay curves were collected for each sample; all lifetimes were analyzed as monoexponential decays. The reported lifetimes are an average of at least three successful and reproducible measurements.

### 3.6. PARACEST and NMR

^1^H NMR spectra and PARACEST measurements were recorded at 400 MHz on a Bruker Advance Spectrometer using a 5 mm BBFO probe in H_2_O/D_2_O (95/5). CEST spectra were recorded at 298 K using a pre-saturation pulse of 3 s at 25 µT (10 mW). The spectra were obtained by recording the bulk water signal intensity as a function of the saturation frequency in a window scale of 300 ppm saturating each 1 ppm. Sample concentrations were [EuL1] = 82 mM, [EuL2] = 54 mM, [EuL3] = 18 mM, [EuL4] = 18 mM, [NdL1] = 90 mM, [NdL2] = 83 mM, [NdL3] = 18 mM, [NdL4] = 17 mM, [YbL1] = 90 mM, [YbL2] = 82 mM, [YbL3] = 16 mM, [YbL4] = 19 mM. The pH values were adjusted by adding small amounts of diluted KOH or HCl vapor.

### 3.7. Enzymatic Assays

Relaxometric enzymatic assays were monitored for GdL4 in 50 mM of HEPES buffer containing 100 mM of NaCl and 10 mM of DTT at pH = 7.4. The concentration of the complex in the tube was 0.94 mM. 6.69 × 10^−3^ U of the caspase-3 enzyme was added to the complex solution.

Urokinase, high molecular weight from human urine, was purchased from Calbiochem (ref. 672081). Relaxometric enzymatic assays were monitored for GdL3 in 50 mM of phosphate buffer containing 7 mg/mL of BSA at pH = 7.5. The concentration of the complex in the tube was 1.2 mM. 500 U of urokinase enzyme was added to the complex solution.

For both solutions, relaxometric kinetic measurements were recorded every 2 min for 30 min, and then every 30 min for 3 h and finally 24 h after reaction began, by measuring the evolution of the longitudinal relaxation time (T_1_) at 20 MHz and 310 K.

Fluorogenic caspase-3 substrate *N*-acetyl-DEVD-AFC was purchased from Sigma-Aldrich (ref. A0466). Fluorescence enzymatic assay was performed in 50 mM HEPES buffer containing 100 mM of NaCl and 10 mM of DTT at pH = 7.4, for 0.7 µM of *N*-acetyl-DEVD-AFC with 0.005 U of caspase-3. The enzymatic assay was monitored at 37 °C by recording fluorescence emission at 460 nm after excitation at 380 nm as a function of time. The caspase-3 inhibition test was recorded in the same conditions by adding increasing concentrations of GdL4 (100 µM and 500 µM).

Urokinase luminescent substrate N-Cbz-GGR-AMC was purchased from Sigma-Aldrich (ref. C9396). The fluorescence enzymatic assay was performed in 50 mM potassium phosphate buffer containing 7 mg/mL of BSA at pH = 7.5 for 0.5 µM of N-Cbz-GGR-AMC with 0.5 U of urokinase. The enzymatic assay was monitored at 37 °C by recording fluorescence emission at 440 nm after excitation at 365 nm as a function of time. The urokinase inhibition test was recorded in the same conditions by adding increasing concentrations of GdL3 (5 µM, 10 µM, 50 µM and 100 µM).

## 4. Conclusions

We report here the synthesis of two DO3A-derivative ligands, L3 and L4, bearing peptide side-chains, which are substrates of urokinase and caspase-3, as well as the physical-chemical characterization of their Ln^3+^ complexes. The synthesis involved the use of backbone amide-linker 5-(4-formyl-3,5-dimethoxyphenoxy)valeric acid, introduced first on the aminomethylated polystyrene resin. This spacer presents the advantage of being labile in acidic conditions and can therefore be removed during the final step of deprotection. This innovative approach allowed us to easily obtain the pure peptide-derivative ligands in excellent yield. The amine-derivative compound mimicking the complex after enzymatic cleavage, LnL1, as well as a model complex without the peptide side chain, LnL2, were also synthesized and studied. The analysis of field-dependent relaxivity data on the Gd^3+^ complexes, combined with luminescence lifetime measurements on the Eu^3+^ analogues, provided insight into the coordination environment of the lanthanide ion. The peptide-derivative complexes are non-hydrated, as the peptide side chains can provide extra coordinating functions to prevent water access to the metal. Nevertheless, the relaxivities remain relatively high for a non-hydrated complex thanks to an important second sphere contribution. A weak PARACEST effect is detected for some of the Nd^3+^, Eu^3+^ or Yb^3+^ chelates, in particular those formed with the L1 ligand bearing a propylamine pendant arm. No enzymatic transformation could be observed for the LnL3 and LnL4 complexes, which is likely related to the involvement of the peptide side chain in metal coordination. These results illustrate again that the prediction of Ln^3+^ coordination modes remains difficult even for widely studied DO3A-type chelates.

## Figures and Tables

**Table 1:** Eu^3+^ luminescence lifetime measurements (pH/pD = 7.4; λ_exc_ = 396 nm and λ_em_ = 616 nm), calculated inner sphere hydration numbers, and relaxivity values of the GdL complexes (pH 7.5, 25 °C and 20 MHz).

	τ_H2O_ (ms) ^1^	τ_D2O_ (ms) ^1^	*q* ^2^	r_1_ (mM^−1^·s^−1^) ^3^
LnL1	0.422 (4)	1.54 (1)	1.6 (3)	8.26
LnL2	0.506 (3)	1.431 (9)	1.0 (3)	4.02
LnL3	0.638 (6)	1.72 (1)	−0.4 (4) ^4^	3.27
LnL4	0.925 (8)	1.64 (1)	0.1 (3)	3.65

^1^ for Ln=Eu complexes. ^2^ Calculated according to equations (1) and (2). ^3^ for Ln=Gd complexes; ^4^ assuming the additional coordination of an amine from the side chain, otherwise *q* = 0.7 (3) is obtained; see text.

**Table 2:** Main parameters obtained from the fitting of the NMRD profiles. Underlined parameters have been fixed. SS stands for second-sphere.

	GdL1	GdL2	GdL3	GdL4
q	2	1	0	0
q^SS^	0	0	1	1
k_ex_^298^ (10^6^ s^−1^)	11 ^a^	111 ^b^	-	-
ΔH^≠^ (kJ·mol^−1^)	33.6 ^a^	21.0 ^b^	-	-
k_ex_^298 SS^ (10^6^ s^−1^)	-	-	2000 ^c^	2000 ^c^
ΔH^≠ SS^ (kJ·mol^−1^)	-	-	30 ^c^	30 ^c^
E_R_ (kJ·mol^−1^)	15 (3)	68 (7)	12 (6)	15 (4)
τ_R_^298^ (ps)	95 (5)	55 (5)	160(10)	260 (20)
r_GdH_ (Å)	3.1	3.1	3.8 ^d^	3.8 ^d^

^a^. fixed to values of GdDO3A, ref. [32]. ^b^. fixed to values of GdL6, ref. [27] ^c^. fixed to values from ref. [33]. ^d^. for second-sphere water protons.

## Data Availability

The data presented in this study are available in Supplementary Material.

## Supplementary Materials

The following are available online. Figure S1: ^1^H NMRD profile of GdL1; Figure S2: ^1^H NMRD profile of GdL2; Figure S3: ^1^H NMRD profile of GdL3; Figure S4: ^1^H NMRD profile of GdL4; Figure S5: ^1^H NMR spectra of YbL3 at 25 °C. Figures S6–S12: Chemical structures and atom numbering scheme of intermediates and final compounds; Equations used for the analysis of the NMRD data; Table S1. Full parameter set obtained from the fitting of the NMRD profiles.

## Sample Availability

Samples of the compounds are not available from the authors.

## References

[1] Soleimany A.P., Bhatia S.N. (2020). Activity-based diagnostics: An emerging paradigm for disease detection and monitoring. Trends Mol. Med..

[2] Wahsner J., Gale E.M., Rodríguez-Rodríguez A., Caravan P. (2018). Chemistry of MRI contrast agents: Current challenges and new frontiers. Chem. Rev..

[3] Hingorani D.V., Yoo B., Bernstein A.S., Pagel M.D. (2014). Detecting enzyme activities with exogenous MRI contrast agents. Chem. Eur. J..

[4] Li H., Meade T.J. (2019). Molecular magnetic resonance imaging with Gd(III)-based contrast agents: Challenges and key advances. J. Am. Chem. Soc..

[5] Kim D., Lee Y.D., Jo S., Kim S., Lee T.S. (2020). Detection and imaging of cathepsin l in cancer cells using the aggregation of conjugated polymer dots and magnetic nanoparticles. Sens. Actuator B Chem..

[6] Korchak S., Jagtap A.P., Glöggler S. (2021). Signal-enhanced real-time magnetic resonance of enzymatic reactions at millitesla fields. Chem. Sci..

[7] Wyskocka-Gajda M., Przypis L., Olesiejuk M., Krawczyk T., Kuznik A., Nawara K., Minoshima M., Sugihara F., Kikuchi K., Kuznik N. (2021). A step towards gadolinium-free bioresponsive MRI contrast agent. Eur. J. Med. Chem..

[8] Eills J., Cavallari E., Kircher R., Di Matteo G., Carrera C., Dagys L., Levitt M.H., Ivanov K.L., Aime S., Reineri F. (2021). Singlet-contrast magnetic resonance imaging: Unlocking hyperpolarization with metabolism **. Angew. Chem.-Int. Edit..

[9] Mason S.D., Joyce J.A. (2011). Proteolytic networks in cancer. Trends Cell. Biol..

[10] Vizovisek M., Ristanovic D., Menghini S., Christiansen M.G., Schuerle S. (2021). The tumor proteolytic landscape: A challenging frontier in cancer diagnosis and therapy. Int. J. Mol. Sci..

[11] Merbach A., Helm L., Toth E. (2013). The Chemistry of Contrast Agents in Medical Magnetic Resonance Imaging.

[12] Vinogradov E., Sherry A.D., Lenkinski R.E. (2013). CEST: From basic principles to applications, challenges and opportunities. J. Magn. Reson..

[13] Woods M., Woessner D.E., Sherry A.D. (2006). Paramagnetic lanthanide complexes as paracest agents for medical imaging. Chem. Soc. Rev..

[14] Hingorani D.V., Bernstein A.S., Pagel M.D. (2015). A review of responsive MRI contrast agents: 2005–2014. Contrast Media Mol. Imaging.

[15] Toth E., Bonnet C.S. (2019). Responsive paracest contrast agents. Inorganics.

[16] Lilley L.M., Kamper S., Caldwell M., Chia Z.K., Ballweg D., Vistain L., Krimmel J., Mills T.A., MacRenaris K., Lee P. (2020). Self-immolative activation of beta-galactosidase-responsive probes for in vivo mr imaging in mouse models. Angew. Chem. Int. Ed..

[17] Sinharay S., Randtke E.A., Howison C.M., Ignatenko N.A., Pagel M.D. (2018). Detection of enzyme activity and inhibition during studies in solution, in vitro and in vivo with catalycest MRI. Mol. Imaging Biol..

[18] Ali M.M., Liu G., Shah T., Flask C.A., Pagel M.D. (2009). Using two chemical exchange saturation transfer magnetic resonance imaging contrast agents for molecular imaging studies. Acc. Chem. Res..

[19] Yoo B., Pagel M.D. (2006). A paracest MRI contrast agent to detect enzyme activity. J. Am. Chem. Soc..

[20] Yoo B., Sheth V.R., Howison C.M., Douglas M.J.K., Pineda C.T., Maine E.A., Baker A.F., Pagel M.D. (2014). Detection of in vivo enzyme activity with catalycest MRI. Magn. Reson. Med..

[21] Sinharay S., Howison C.M., Baker A.F., Pagel M.D. (2017). Detecting in vivo urokinase plasminogen activator activity with a catalycest MRI contrast agent. NMR Biomed..

[22] Chauvin T., Durand P., Bernier M., Meudal H., Doan B.T., Noury F., Badet B., Beloeil J.C., Toth E. (2008). Detection of enzymatic activity by paracest MRI: A general approach to target a large variety of enzymes. Angew. Chem. Int. Ed..

[23] Chauvin T., Torres S., Rosseto R., Kotek J., Badet B., Durand P., Toth E. (2012). Lanthanide(III) complexes that contain a self-immolative arm: Potential enzyme responsive contrast agents for magnetic resonance imaging. Chem. Eur. J..

[24] He J.F., Bonnet C.S., Eliseeva S.V., Lacerda S., Chauvin T., Retailleau P., Szeremeta F., Badet B., Petoud S., Toth E. (2016). Prototypes of lanthanide(III) agents responsive to enzymatic activities in three complementary imaging modalities: Visible/near-infrared luminescence, paracest-, and T_1_-MRI. J. Am. Chem. Soc..

[25] Zeng Q., Zhang R., Zhang T., Xing D. (2019). H_2_O_2_-responsive biodegradable nanomedicine for cancer-selective dual-modal imaging guided precise photodynamic therapy. Biomaterials.

[26] Redy O., Kisin-Finfer E., Sella E., Shabat D. (2012). A simple fret-based modular design for diagnostic probes. Org. Biomol. Chem..

[27] Congreve A., Parker D., Gianolio E., Botta M. (2004). Steric control of lanthanide hydration state: Fast water exchange at gadolinium ion in a mono-amide dota complex. Dalton Trans..

[28] Yoo B., Pagel M.D. (2006). A facile synthesis of a-amino-dota as a versatile molecular imaging probe. Tet. Lett..

[29] Yang Y. (2016). Side Reactions in Peptide Synthesis. Peptide Racemization.

[30] Yoo B., Sheth V.R., Pagel M.D. (2009). An amine-derivatized, dota-loaded polymeric support for Fmoc solid phase peptide synthesis. Tet. Lett..

[31] Supkowski R.M., Horrocks D. (1999). Displacement of inner-sphere water molecules from Eu(III) analogues of Gd(III) MRI contrast agents by carbonate and phosphonate anions: Dissociation constants from luminescence data in the rapid-exchange limit. Inorg. Chem..

[32] Toth E., Ni Dhubhghaill O.M., Besson G., Helm L., Merbach A.E. (1999). Coordination equilibrium—A clue for fast water exchange on potential magnetic resonance imaging contrast agents?. Magn. Reson. Chem..

[33] Lebduskova P., Hermann P., Helm L., Toth E., Kotek J., Binnemans K., Rudovsky J., Lukes I., Merbach A.E. (2007). Gadolinium(III) complexes of mono- and diethyl esters of monophosphonic acid analogue of dota as potential MRI contrast agents: Solution structures and relaxometric studies. Dalton Trans..

[34] Krchová T., Kotek J., Jirák D., Havlíčková J., Císařová I., Hermann P. (2013). Lanthanide(III) complexes of aminoethyl-do3a as paracest contrast agents based on decoordination of the weakly bound amino group. Dalton Trans..

[35] Aime S., Barge A., Castelli D.D., Fedeli F., Mortillaro A., Nielsen F.U., Terreno E. (2002). Paramagnetic lanthanide(III) complexes as ph-sensitive chemical exchange saturation transfer (CEST) contrast agents for MRI applications. Magn. Reson. Med..

[36] Terreno E., Delli Castelli D., Cravotto G., Milone L., Aime S. (2004). Ln(III)-dotamgiy complexes: A versatile series to assess the determinants of the efficacy of paramagnetic chemical exchange saturation transfer agents for magnetic resonance imaging applications. Investig. Radiol..

[37] Aime S., Botta M., Fasano M., Marques M.P.M., Geraldes C.F.G.C., Pubanz D., Merbach A.E. (1997). Conformational and coordination equilibria on dota complexes of lanthanide metal ions in aqueous solution studied by 1h nmr spectroscopy. Inorg. Chem..

