# Supplementary material for: Lanthanide DO3A-Complexes Bearing Peptide Substrates: The Effect of Peptidic Side Chains on Metal Coordination and Relaxivity
Source: Molecules. 2021 Apr 9;26(8):2176. doi: 10.3390/molecules26082176 (PMC8069257; doi:10.3390/molecules26082176)
Supplement: Supplementary file 1 [file molecules-26-02176-s001.pdf]

molecules

Article

Lanthanide DO3A-Complexes Bearing Peptide Substrates: The Effect of Peptidic Side Chains on Metal Coordination and Relaxivity

Sophie Laine, Jean-François Morfin, Mathieu Galibert, Vincent Aucagne, Célia S. Bonnet and Éva Tóth *

Centre de Biophysique Moléculaire, CNRS UPR 4301, Rue Charles Sadron, CEDEX 2, 45071 Orléans, France; laine-sophie@laposte.net (S.L.); jmorfin@cnrs-orleans.fr (J.-F.M.); mathieu.galibert@genepep.com (M.G.); vincent.aucagne@cnrs-orleans.fr (V.A.); celia.bonnet@cnrs.fr (C.S.B.)

* Correspondence: eva.jakabtoth@cnrs-orleans.fr; Tel.: +33-2-38-25-76-25

Synthetic procedures

Figure S1 : ^{1}H NMRD profile of GdL1

Figure S2 : ^{1}H NMRD profile of GdL2

Figure S3 : ^{1}H NMRD profile of GdL3

Figure S4 : ^{1}H NMRD profile of GdL4

Table S1. Full parameter set obtained from the fitting of the NMRD profiles.

Figure S5. ^{1}H NMR spectra of YbL3 at 25°C.

Figure S6-S12. Chemical structures and atom numbering scheme of intermediates and final compounds

Equations used for the analysis of the NMRD data

Synthesis

Tri-tert-butyl 2, 2′, 2′′-(10-(3-(1, 3-dioxoisoindolin-2-yl)propyl)-1, 4, 7, 10- tetraazacyclododecane-1, 4, 7-triyl) tri-acetate : 2

DO3AtBu (500 mg, 0.97 mmol) was dissolved in acetonitrile (30 mL). Sodium iodide (100 mg, 0.67 mmol), *N*-(3-bromo-propyl)phtalimide (311 mg, 1.16 mmol) and K_2CO_3 (421 mg, 3.05 mmol) were added to the solution, and the mixture was stirred 3 days at 80°C. The solids were filtered off and the solvent was evaporated. The crude product was purified by flash chromatography (CH_2Cl_2/MeOH 9:1) to give 475 mg of **2** as a yellow oil with a yield of 70%.

^{1}H NMR: ($CDCl_3$, 600 MHz, δ ppm) = 1.23-1.26 (27H, bs, H12,14,18); 1.63 (2H, tt, $^3J_{H22-H23}$= $^3J_{H22-H21}$=7.6 Hz, H22); 2.09-2.79 (18H, m, H1,2,3,4,5,6,7,8,21); 2.83-3.11 (6H, m, H9,15,19); 3.48 (2H, t, ^{3}J=7.6 Hz, H23); 7.59-7.64 (4H, m, H27,28,29,30).

^{13}C NMR: ($CDCl_3$, 150 MHz, δ ppm) =25.8 (C22); 27.9, 28.0, 28.2 (C18, 12, 14), 36.2 (C23); 50.6 (C21); 51.2, 51.9, 53.0, 53.1 (C1,2,3,4,5,6,7,8); 55.9, 57.1 (C9,15,19); 82.1, 82.5, 82.9 (C11,13,17); 123.4, (C27,30); 132.1(C26,31); 134.3 (C28,29); 168.3 (C24,25); 169.9, 172.9 (C10,16,20).

HRMS: calculated for $C_{37}H_{60}N_5O_8$ $[M+H]^+$: *m/z*= 702.4436, found $[M+H]^+$: *m/z*= 702.4433

Tri-tert-butyl 2, 2′, 2′′-(10-(3-Aminopropyl)-1, 4, 7, 10-tetraazacyclododecane-1, 4, 7- triyl) triacetate : 3

Compound **2** (569 mg, 0.811mmol) was dissolved in hydrazine hydrate (30 mL). The reaction mixture was stirred at 90 °C during 30 min. Hydazine was evaporated and the residue was dissolved in dichloromethane, and washed several times with water. The organic phase was then dried over sodium sulfate and the solvent was evaporated to give 278 mg of the compound **4**, as a yellowish powder with a 60% yield.

^{1}H NMR: ($CDCl_3$, 600 MHz, δ ppm) = 1.40 (18H, s, H18, 20); 1.41 (9H, s, H19); 1.59 (2H, tt, $^3J_{H22-H23}$= $^3J_{H22-H21}$=5.2Hz, H22); 2.52 (18H, m, H1,2,3,4,5,6,7,8,21); 3.12 (8H, bs, H9,10,11,23); 8.19 (2H, s, NH2).

^{13}C NMR: ($CDCl_3$, 150 MHz, δ ppm) = 23.6 (C22); 27.8 (C19); 27.9 (C18,20); 39.2 (C23); 49.6(C21); 50.0 (C4,5); 50.8 (C1,2,3,6,7,8); 56.6 (C10,11); 57.9 (C9); 81.8 (C15,17); 88.5 (C16); 170.5 (C14,12); 172.7 (C13).

HRMS: calculated for $C_{29}H_{58}N_5O_6$ $[M+H]^+$: *m/z*=572.4381, found $[M+H]^+$: *m/z*= 572.4381

2, 2′, 2′′-(10-(3-aminopropyl)-1, 4, 7, 10-tetraazacyclododecane-1, 4, 7-triyl) triacetic acid : L1

To a solution of compound **3** (154 mg, 0.27 mmol) in dichloromethane (6 mL), was added trifluoroacetic acid (6.22 mL, 81 mmol). The reaction mixture was stirred 18 h at room temperature. The product was then evaporated under vacuum. The resulting oil was dissolved in small amount of dichloromethane and cold diethyl ether was added. The precipitate was filtered and dried under vacuum to give 60 mg of **L1** as a white powder with a 55% yield.

^{1}H NMR: (D_2O, 600 MHz, δ ppm) = 1.80 (2H, bs, H16); 2.3 (2H, bs, H15); 2.92-3.24 (16H, m, H1,2,3,4,5,6,7,8); 3.32-3.77 (8H, m, H9,11,13,17).

^{13}C NMR: (D_2O, 150 MHz, δ ppm) = 25.5 (C16); 36.6 (C17); 48.5, 49.5, 50.3, 51.1 (C1,2,3,4,5,6,7,8); 53.7 (C15); 56.1 (C9,11,13); 170.8 (C10); 173.7 (C12,14).

HRMS: calculated for $C_{17}H_{34}N_5O_6$ $[M+H]^+$: *m/z*=404.2503, found $[M+H]^+$: *m/z*= 404.2504

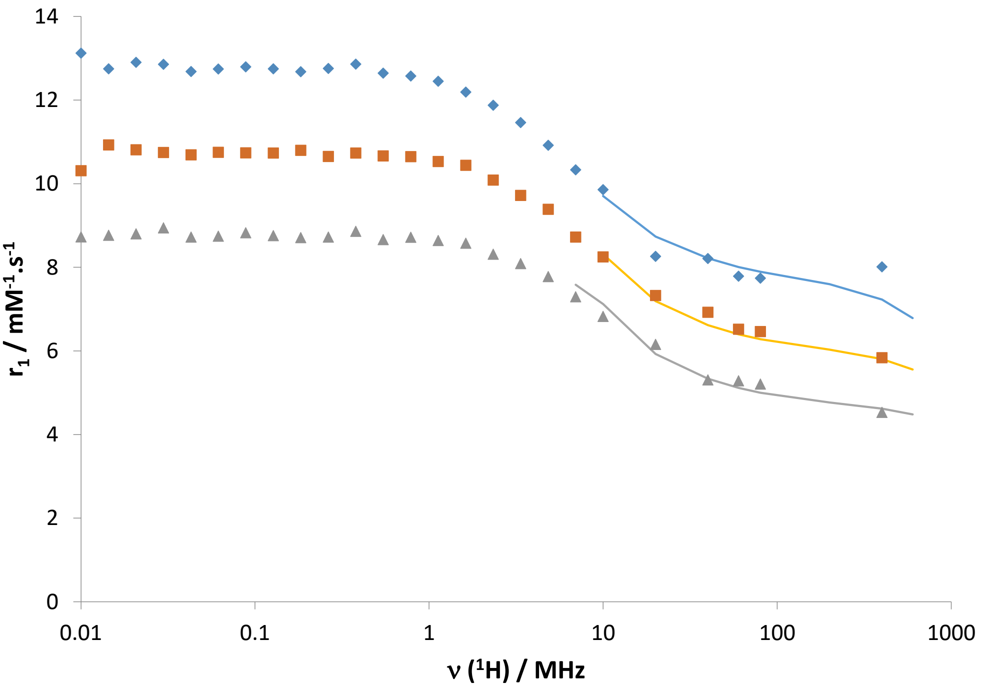

Figure S1 : ^{1}H NMRD profile of GdL1 6.39 mM, at pH = 7 at 25°C (◆), 37°C (■), and 50°C (▲). The line corresponds to the best fit to the SBM equations with the parameters in Table S1

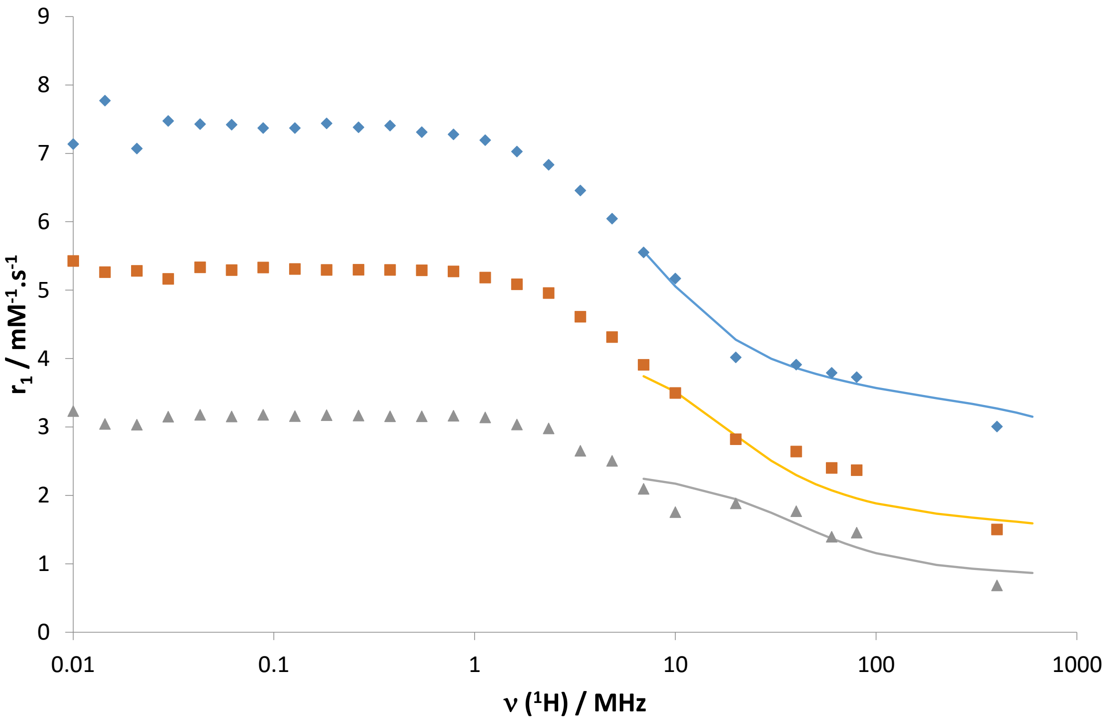

Figure S2 : ^{1}H NMRD profile of GdL2 1.23 mM, at pH = 7.17 at 25°C (◆), 37°C (■), and 50°C (▲).The line corresponds to the best fit to the SBM equations with the parameters in Table S1

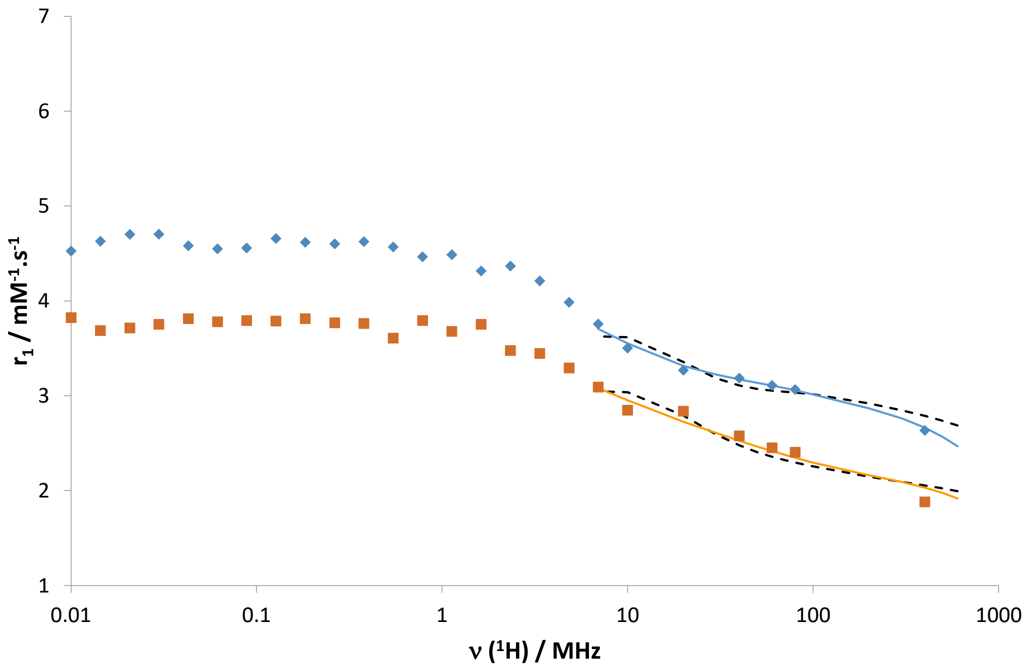

Figure S3 : ^{1}H NMRD profile of GdL3 0.96 mM, at pH = 7.36 at 25°C (◆), 37°C (■), and 50°C (▲). The dotted line corresponds to the best fit to the SBM equations with the parameters in Table S1 corresponding to *q* = 1 (one inner-sphere water molecule), while the solid line represents the parameters corresponding to a second sphere contribution with q^{SS} = 1.

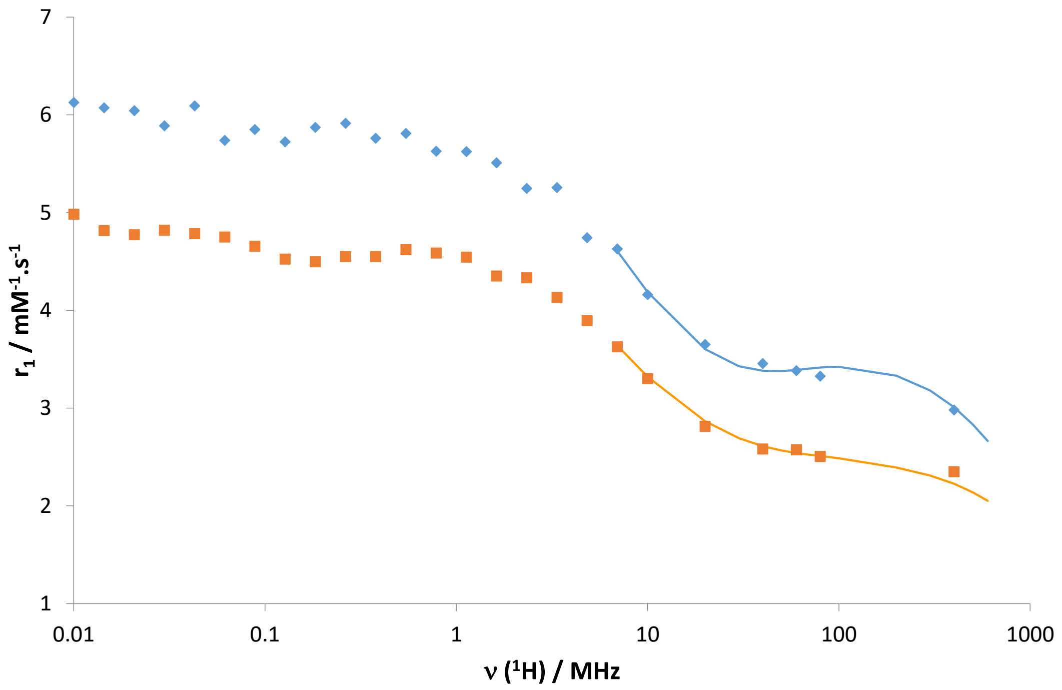

Figure S4 : ^{1}H NMRD profile of GdL4 0.93 mM, at pH = 6.98 at 25°C (◆), 37°C (■), and 50°C (▲).The line corresponds to the best fit to the SBM equations with the parameters in Table S1

Table S1. Full parameter set obtained from the fitting of the NMRD profiles. Underlined parameters have been fixed.

	**GdL1**	**GdL2**	**GdL3**		**GdL4**
q	2	1	1	0	0
q^{SS}	-	-	-	1	1
k_{ex}^{298} ($10^6 s^{-1}$)	11	111	111	-	-
$\Delta H^{\neq}$ ($kJ.mol^{-1}$)	33.6	21.0	21.0	-	-
$k_{ex}^{298\ SS}$ ($10^6 s^{-1}$)	-	-	-	2000	2000
$\Delta H^{\neq\ SS}$ ($kJ.mol^{-1}$)	-	-	-	30	30
E_R ($kJ.mol^{-1}$)	15 (3)	68 (7)	15 (5)	12 (6)	15 (4)
τ_R^{298} (ps)	95 (5)	55 (5)	49 (4)	160(10)	260 (20)
D_{GdH}^{298}(10^{-9} $m^2 s^{-1}$)	26	26	26	26	26
E_{DGdH} ($kJ.mol^{-1}$)	19 (7)	30 (3)	27 (4)	23 (4)	26 (2)
τ_v^{298}(ps)	100 (20)	18 (9)	16 (3)	19 (4)	4.4 (7)
Δ^2($10^{20} s^{-1}$)	0.7 (2)	0.7 (2)	2.7 (3)	1.14 (9)	0.8 (1)
r_{GdH}(Å)	3.1	3.1	3.8	3.8	3.8

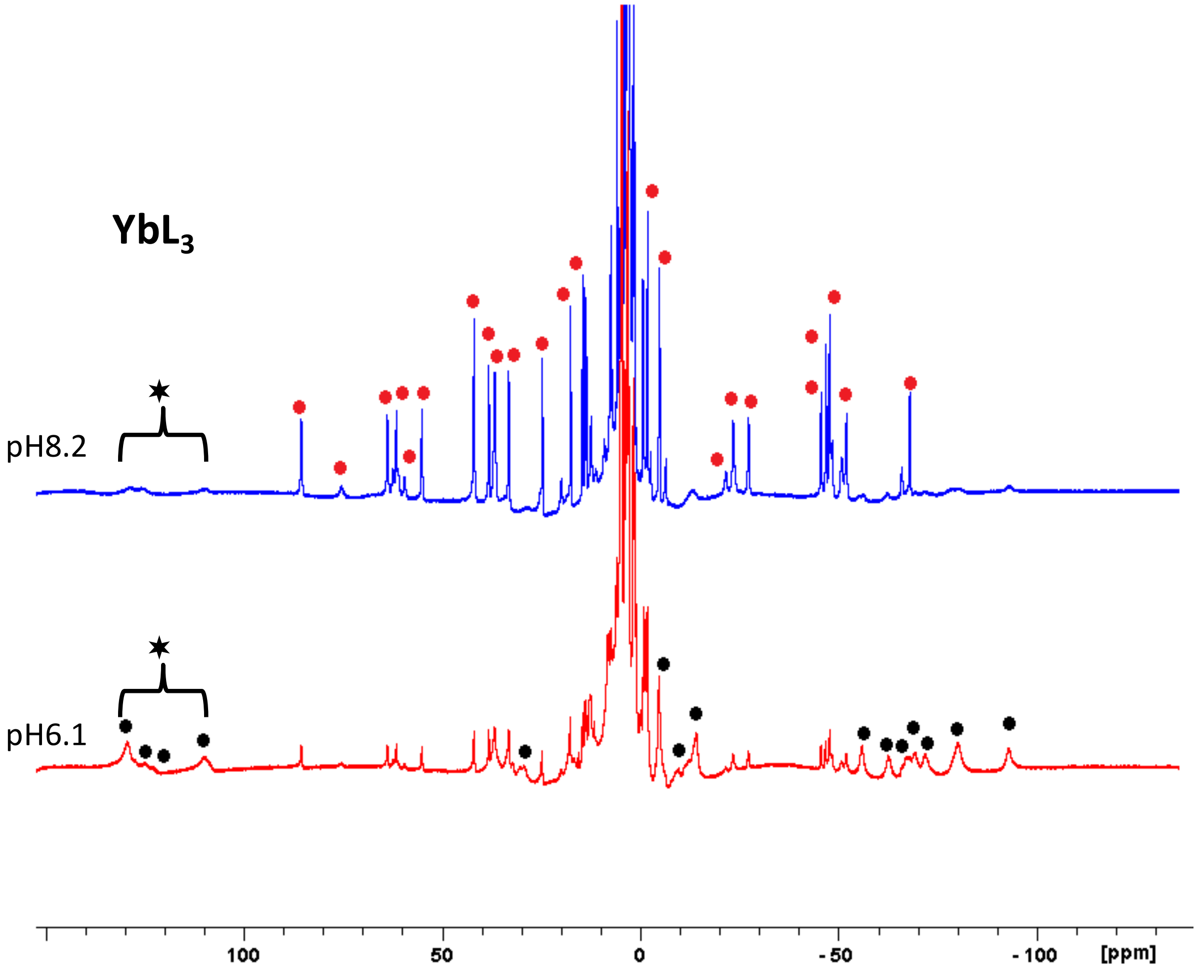

Figure S5. ^{1}H NMR spectra of YbL3 at 25°C. Peaks that can be attributed to the major isomer are indicated by ● in the spectrum recorded at pH 8.2 and by ● in the spectrum recorded at pH 6.1. ✶ represents the axial protons of the macrocycle belonging to the SAP isomer.

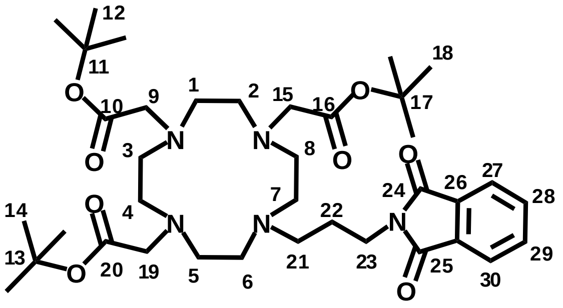

Figure S6. Chemical structure and atom numbering scheme of **2**

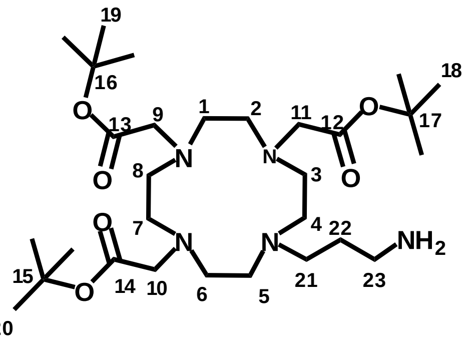

Figure S7. Chemical structure and atom numbering scheme of **3**

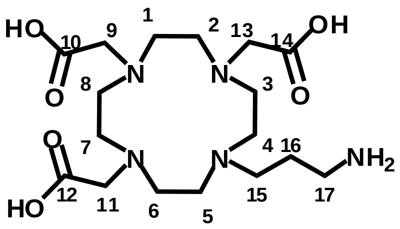

Figure S8. Chemical structure and atom numbering scheme of **L1**

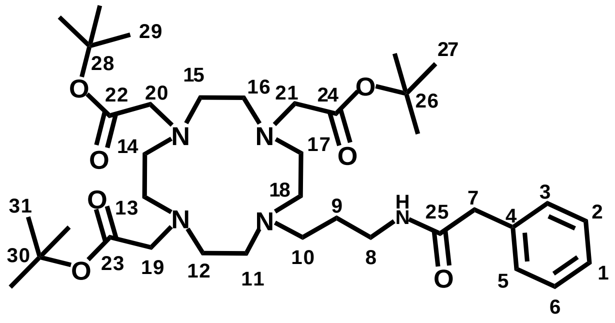

Figure S9. Chemical structure and atom numbering scheme of protected **L2**

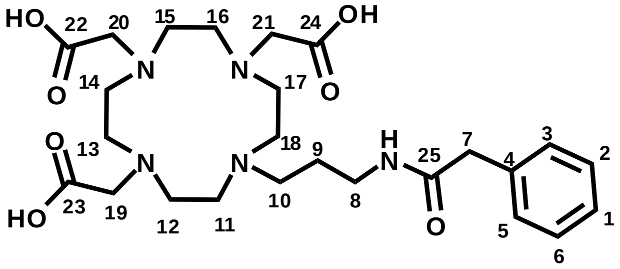

Figure S10. Chemical structure and atom numbering scheme of **L2**

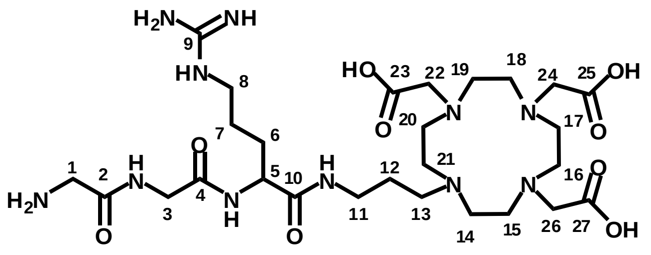

Figure S11. Chemical structure and atom numbering scheme of **L3**

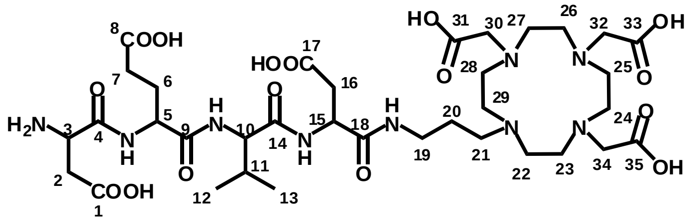

Figure S12. Chemical structure and atom numbering scheme of **L4**

Equations used for the analysis of the NMRD data

The measured longitudinal proton relaxation rate, R_1^{obs} is the sum of the paramagnetic and diamagnetic contributions as expressed in Eq. 1, where r_1 is the proton relaxivity:

$$R_1^{obs} = R_1^d + R_1^p = R_1^d + r_1 \times c_{Gd} \qquad [1]$$

The relaxivity can be divided into terms of inner, second and outer sphere, as follows:

$$r_1 = r_{1IS} + r_{1SS} + r_{1OS} \qquad [2]$$

The inner sphere term is obtained in Eq. 3, where q is the number of inner sphere water molecules.[1]

$$r_{1is} = \frac{1}{1000} \times \frac{q}{55.55} \times \frac{1}{T_{1m}^H + \tau_m} \qquad [3]$$

The longitudinal relaxation rate of inner sphere protons, $1/T_{1m}{}^H$ is expressed by Eq. 4, where r_{GdH} is the effective distance between the electron charge and the ^{1}H nucleus, ω_I is the proton resonance frequency and ω_S is the Larmor frequency of the Gd^{III} electron spin.

$$\frac{1}{T_{1m}^H} = \frac{2}{15}\left(\frac{\mu_0}{4\pi}\right)^2 \frac{\hbar^2 \gamma_I^2 \gamma_S^2}{r_{GdH}^6} S(S+1) \times \left[3J(\omega_I;\tau_{d1}) + 7J(\omega_S;\tau_{d2})\right] \qquad [4]$$

$$\frac{1}{\tau_{di}} = \frac{1}{\tau_m} + \frac{1}{\tau} + \frac{1}{T_{ie}} \qquad [5]$$

The longitudinal and transverse electronic relaxation rates, $1/T_{1e}$ and $1/T_{2e}$ are expressed by Eq. 6-7, where τ_V is the electronic correlation time for the modulation of the zero-field-splitting interaction, E_V the corresponding activation

energy and Δ^2 is the mean square zero-field-splitting energy. We assumed a simple exponential dependence of τ_V versus $1/T$.

$$\left(\frac{1}{T_{1e}}\right)^{ZFS} = \frac{1}{25}\Delta^2\tau_v\{4S(S+1)-3\}\left(\frac{1}{1+\omega_S^2\tau_v^2}+\frac{4}{1+4\omega_S^2\tau_v^2}\right) \quad [6]$$

$$\left(\frac{1}{T_{2e}}\right)^{ZFS} = \Delta^2\tau_v\left(\frac{5.26}{1+0.372\,\omega_S^2\tau_v^2}+\frac{7.18}{1+1.24\omega_S\tau_v}\right) \quad [7]$$

$$\tau_v = \tau_v^{298}\, exp\left\{\frac{E_v}{R}\left(\frac{1}{T}-\frac{1}{298.15}\right)\right\} \quad [8]$$

The second sphere contribution is described as the inner sphere contribution with adapted parameters, in particular r_{GdH}.

The outer-sphere contribution can be described by Eq. 9 where N_A is the Avogadro constant, and J_{os} is its associated spectral density function.[2],[3]

$$r_{1os} = \frac{32N_A\pi}{405}\left(\frac{\mu_0}{4\pi}\right)^2\frac{\hbar^2\gamma_S^2\gamma_I^2}{a_{GdH}D_{GdH}}S(S+1)\left[3J_{os}(\omega_I,T_{1e})+7J_{os}(\omega_S,T_{2e})\right] \quad [9]$$

$$J_{os}(\omega,T_{je}) = Re\left[\frac{1+\frac{1}{4}\left(i\omega\tau_{GdH}+\frac{\tau_{GdH}}{T_{je}}\right)^{1/2}}{1+\left(i\omega\tau_{GdH}+\frac{\tau_{GdH}}{T_{je}}\right)^{1/2}+\frac{4}{9}\left(i\omega\tau_{GdH}+\frac{\tau_{GdH}}{T_{je}}\right)+\frac{1}{9}\left(i\omega\tau_{GdH}+\frac{\tau_{GdH}}{T_{je}}\right)^{3/2}}\right] \quad j = 1, 2 \quad [10]$$

The diffusion coefficient for the diffusion of a water proton away from a Gd^{III} complex, D_{GdH}, is assumed to obey an exponential law versus the inverse of the temperature, with an activation energy E_{GdH}, as given in Eq. 11 D_{GdH}^{298} is the diffusion coefficient at 298.15 K.

$$D_{\mathrm{GdH}} = D_{\mathrm{GdH}}^{298} \exp\left\{ \frac{E_{\mathrm{GdH}}}{R} \left(\frac{1}{298.15} - \frac{1}{T} \right) \right\} \qquad [11]$$

[1] Z. Luz, S. Meibomm, *J.Am.Chem.Soc.* **1964**, *86*, 4766-4768.
[2] J. H. Freed, *J. Chem. Phys.* **1978**, *68*, 4034-4037.
[3] S. H. Koenig, R. D. Brown Iii, *Prog. Nucl. Magn. Reson. Spectrosc.* **1990**, *22*, 487-567.
